# Supplementary material for: Immobilized WNT Proteins Act as a Stem Cell Niche for Tissue Engineering
Source: Stem Cell Reports. 2016 Jul 12;7(1):126–37. doi: 10.1016/j.stemcr.2016.06.004 (PMC4944585; doi:10.1016/j.stemcr.2016.06.004)
Supplement: Document S1. Figures S1–S4 [file mmc1.pdf]

Stem Cell Reports, Volume 7

Supplemental Information

Immobilized WNT Proteins Act as a Stem Cell Niche for Tissue Engineering

Molly Lowndes, Michael Rotherham, Joshua C. Price, Alicia J. El Haj, and Shukry J. Habib

Supplemental Figures

Supplementary Figure 1. Long-term storage controls with Comma-β-Geo cells and mESCs, Related to Figure 2

(A) Comma-Dβ-Geo cells seeded onto surfaces immobilized with WNT3A or vehicle. Surfaces were freshly printed or printed then dehydrated and stored at 4°C for 4 months before cell seeding. After 24hrs the number of GFP+ cells per condition determined, summarized in table (below)

(B) Comma-Dβ-Geo cells seeded onto freshly immobilized WNT3A/vehicle or immobilized WNT3A surfaces incubated at 37°C for 7hrs +/- serum. Table below summarizes number of GFP+ cells per condition. The non-bound WNT fractions (from freshly printed or after incubation at 37°C for 7hrs +/- serum) were added to Comma-Dβ-Geo cells seeded onto vehicle surfaces (right).

(C) Representative images of GFP and mCherry expression of Comma-Dβ-Geo cells seeded onto freshly immobilized WNT3A or vehicle surfaces and visualized after 144hrs.

(D) Representative GFP images of Comma-Dβ-Geo cells seeded onto surfaces immobilized with vehicle or WNT3A. An image taken every 24hrs for up to 168hrs and medium was changed every other day. (i) Cells seeded onto vehicle surface. (ii) Cells seeded onto WNT3A surface. (iii) Cells seeded onto vehicle surface after being grown on WNT3A surface for 24hrs. (iv) Cells seeded onto vehicle surface with soluble WNT3A added the first day to the medium. The original medium was kept and further supplemented with a fresh medium without WNT3A (final volume of 200 μl) to keep samples from drying out (v) Cells seeded onto vehicle surface alone

after being grown for 24hrs on vehicle surface with soluble WNT3A supplemented medium.

(E) Percent GFP$^+$ cells on immobilized surfaces stored at 4°C for 14 days (WNT3A ± DTT) determined using automated protocol generated in Volocity software; based on finding overlap of mCherry and GFP objects. (two independent experiments, mean ± SD; statistical significance determined with one-way ANOVA, p values correspond to *<0.05).

(F) The number of GFP$^+$ objects/ESC cell cluster was monitored every 24hrs and upon passaging onto freshly immobilized surfaces. The number of GFP$^+$ objects per cell cluster was significantly different between the two surfaces, (two independent experiments; statistical significance determined with a two-way ANOVA; p values correspond to **<0.01, ***<0.001).

The scale bar represents 50 μM.

Supplementary Figure 2. FACS analysis of WNT responsiveness, Related to Figure 2

FACS analysis of Comma Db-Geo (7x-GFP/SV-40mCherry) cells seeded onto different surfaces for 24hrs.

(A) All conditions were gated for cells (FSC-AxSSC-A), Live (FSC-WxDapi) and single cells (FSC-WxFSC-A).

(B) Control cells used for compensation controls.

(C) Comma Db-Geo (7x-GFP/SV-40mCherry) alone

(D) Comma Db-Geo (7x-GFP/SV-40mCherry) with soluble WNT3A (50ng/mL)

(E) Comma Db-Geo (7x-GFP/SV-40mCherry) seeded onto a BSA coated surface.

(F) Comma Db-Geo (7x-GFP/SV-40mCherry) seeded onto a DTT treated WNT3A surface.

(G) Comma Db-Geo (7x-GFP/SV-40mCherry) seeded onto a WNT3A surface.

Plots are split into 4 quadrants and a FITC-low population is highlighted based on control groups.

Supplementary Figure 3. Additional analysis of marker expression for Comma Dβ-Geo cells and mESCs, Related to Figure 3

(A-F) Single cell fluorescence intensity measurements of Comma Dβ-Geo cells for each marker [SCA1-APC (A and D), 7TCF-GFP (B and E) and SV40-mCherry (C and F)] were combined (three independent experiments) and plotted as a histogram (A-C) and Tukey box plot (D-F) (>1000 cells per experiment with outliers removed from each individual experiment, Q=0.1%). Statistically significant changes in intensity distributions between the two populations were determined using the Kruskal-Wallis test and the Kolmogorov-Smirnov D value is reported.

(G) The expression levels were split into 2-3 categories; corresponding percentages and number of cells analyzed are summarized in a table.

(H) After being grown for two days in ESC medium containing FBS +LIF mESCs on immobilized WNT3A ± DTT or BSA surfaces (supplemented with 50ng/mL soluble WNT3A) ESCs were fixed and stained for alkaline phosphatase (P1) or trypsinized, collected and re-seeded onto freshly immobilized surfaces. After an additional two days the cells were fixed and stained with alkaline phosphatase and quantified (P2). (n=3 biological replicates; statistical significance determined with one-way ANOVA for just the ALP high percentages between surface treatments; p values correspond to *<0.05, **<0.01, ***<0.001)

(I) Representative brightfield and GFP images of Nanog-Venus mESCs grown on WNT3A surfaces (+/- DTT treatment) for 72 hrs. Fluorescence intensity determined using Volocity software and the number of colonies manually. Colonies not above threshold considered Nanog low while colonies above the threshold were considered Nanog high. Population percentages plotted as part of whole for each condition. Colony analysis showed 5% of the colonies on WNT3A DTT surfaces with high Nanog-Venus expression compared to WNT3A surfaces with 28% of the colonies with high Nanog-Venus expression.

Supplementary Figure 4. Raw numbers and representative images of quantified data for hMSC 3D culture, Related to Figure 4

(A) hMSCs cultured on the active WNT3A surfaces coated with collagen gel were stained for DAPI to determine cell number. Gels were imaged as z-stacks and the number of cells in each layer was counted: lower (up to 72μm / 46% gel), middle (up to 132μm, 85% gel) and upper layers (up to 179μm, 100% gel). Values represent average cell counts, error bars represent SEM, * denotes $p<0.05$.

(B) Representative fluorescent immunostaining images from each defined layer. Gels stained for STRO1 (green), Osteocalcin (red) and DAPI (blue) of hMSC in collagen gels after 7 days in culture on WNT3A ± DTT. Merged images show spatial pattern of staining in relation to cell nuclei. Scale bar represents 100 μm.

(C) Representative confocal images (4x magnification) of STRO1 staining at the base of the collagen gel (7 days in culture) in the middle of the well. The scale bar represents 500 μm.

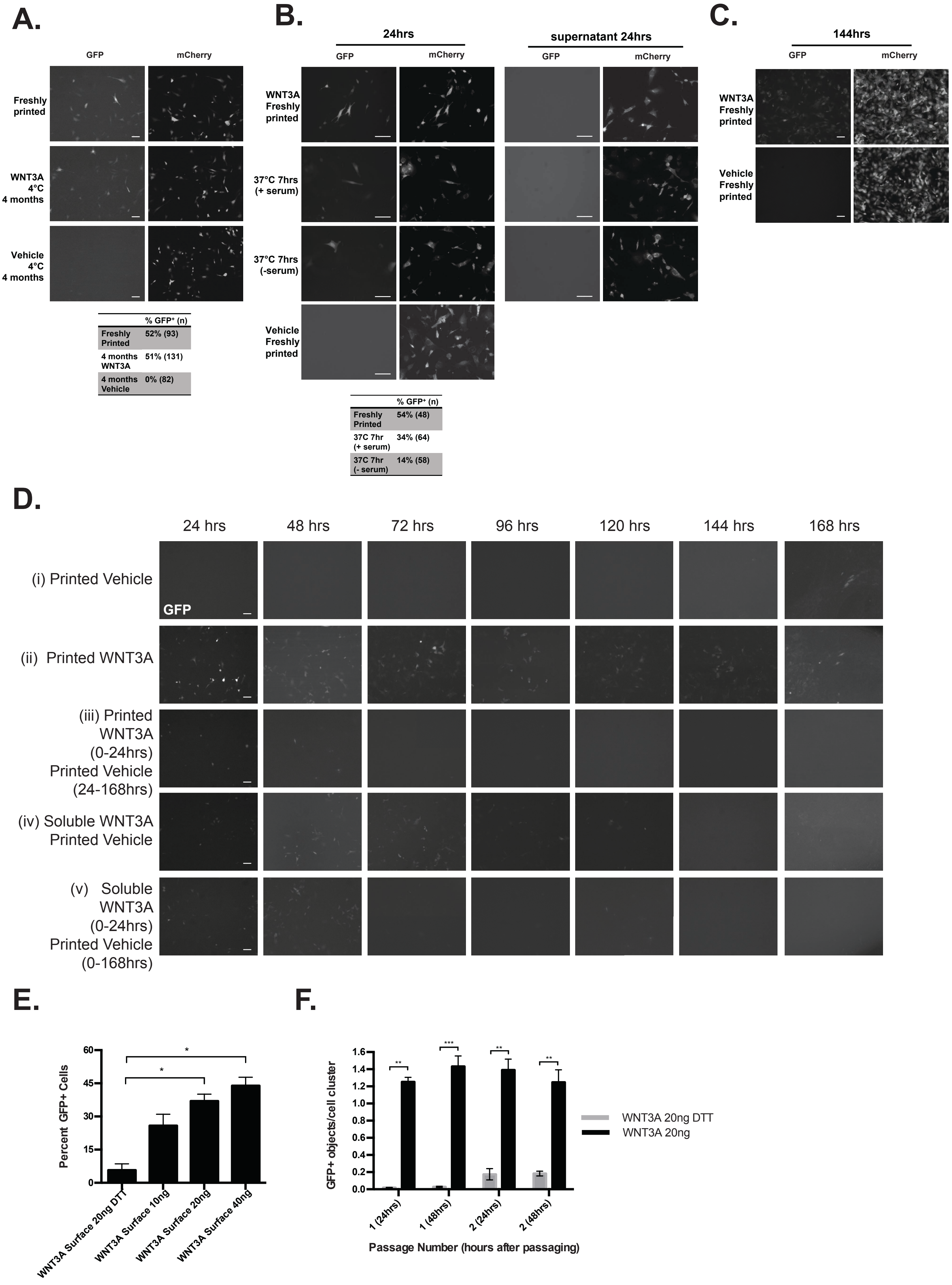

	% GFP+ (n)
Freshly Printed	52% (93)
4 months WNT3A	51% (131)
4 months Vehicle	0% (82)

	% GFP+ (n)
Freshly Printed	54% (48)
37C 7hr (+ serum)	34% (64)
37C 7hr (- serum)	14% (58)

Lowndes et. al. Supplemental Figure 1

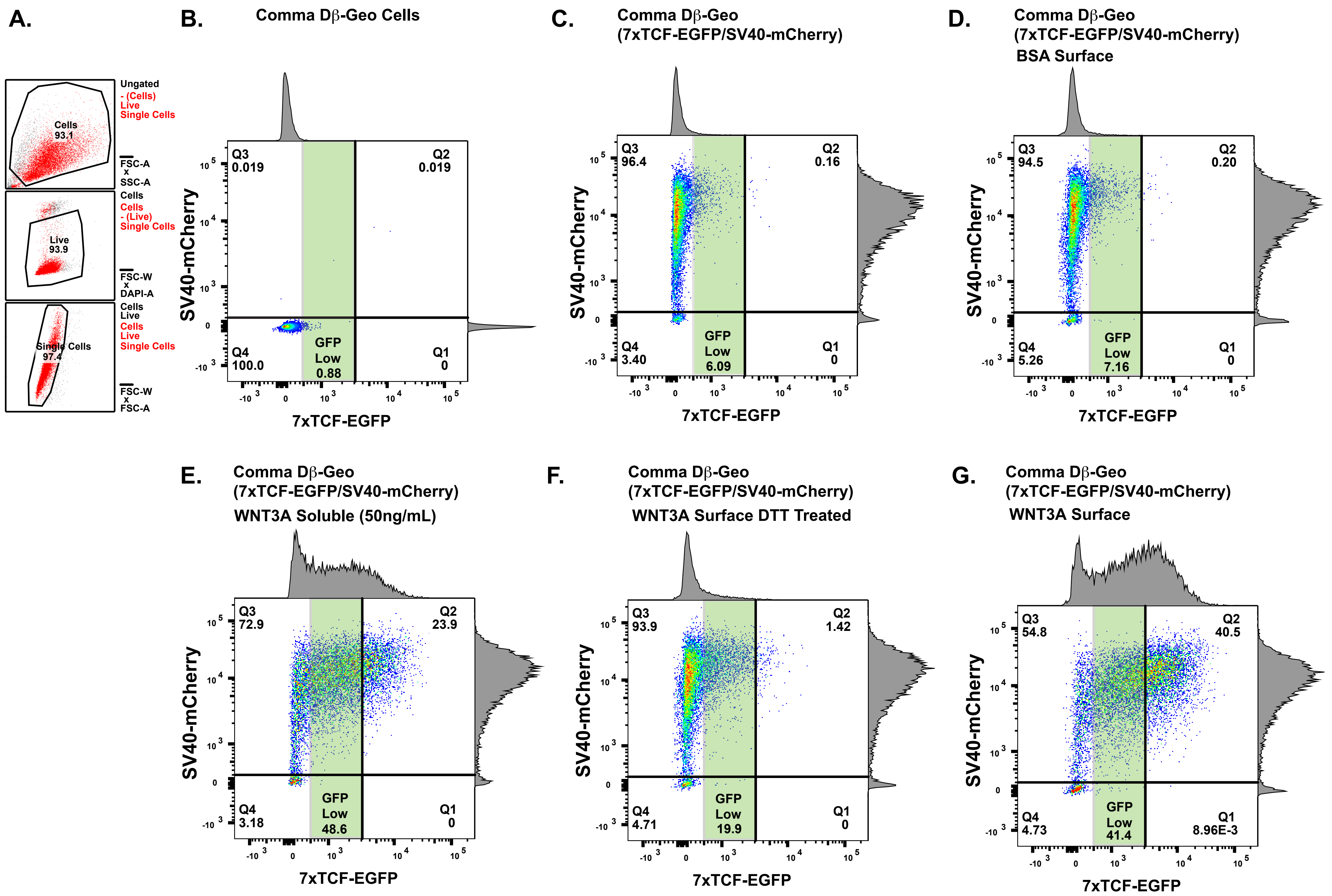

Lowndes et. al. Supplemental Figure 2

A.

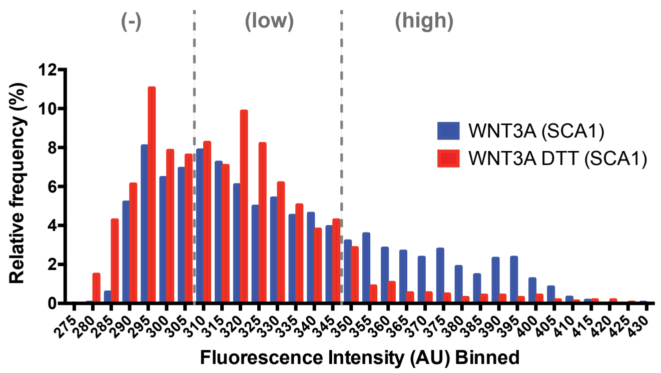

D.

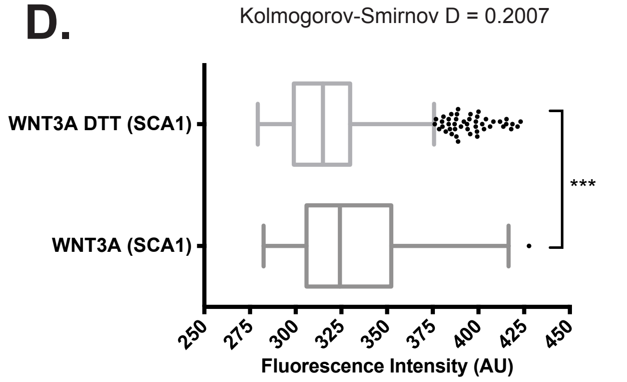

B.

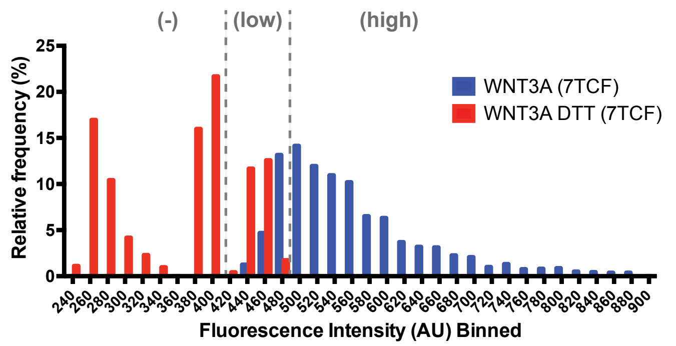

E.

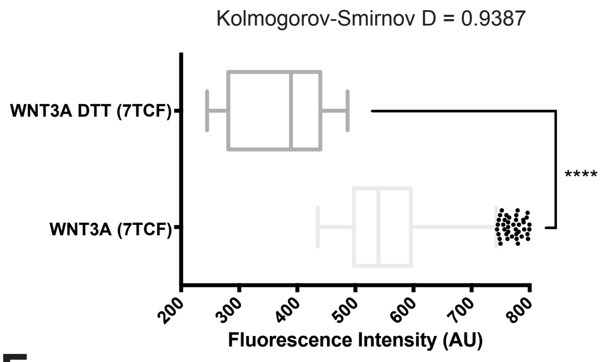

C.

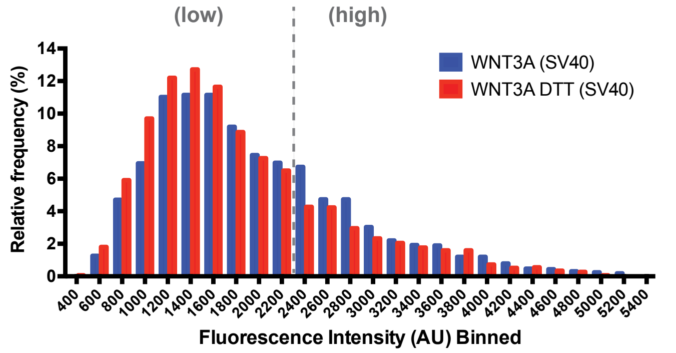

F.

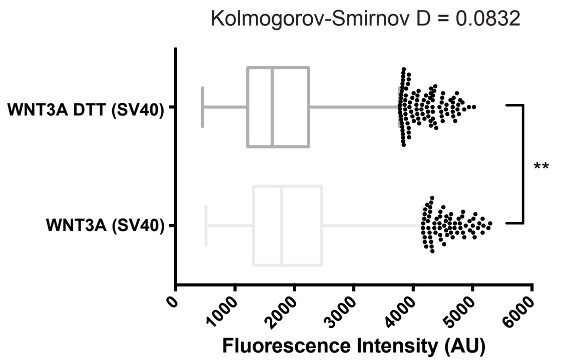

G.

	SCA1 (-)	SCA1 (low)	SCA1 (high)	n
WNT3A DTT	38%	53%	9%	1683
WNT3A	27%	45%	28%	1906

	7TCF (-)	7TCF (low)	7TCF (high)	n
WNT3A DTT	74%	26%	0%	1439
WNT3A	0%	23%	77%	1597

	SV40 (low)	SV40 (high)	n
WNT3A DTT	81%	19%	2877
WNT3A	77%	23%	3166

H.

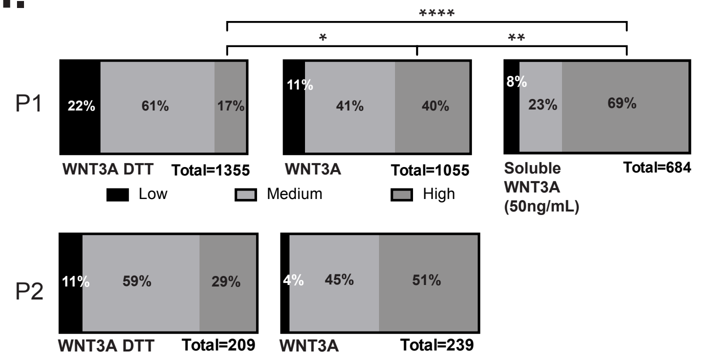

I.

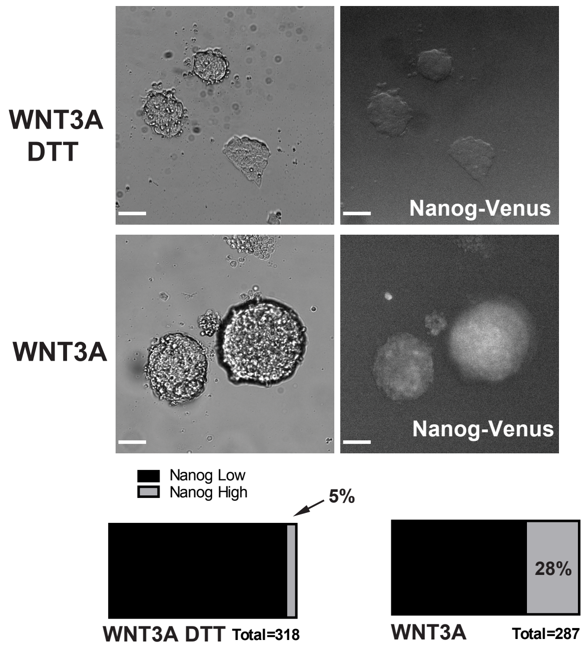

Lowndes et. al. Supplemental Figure 3

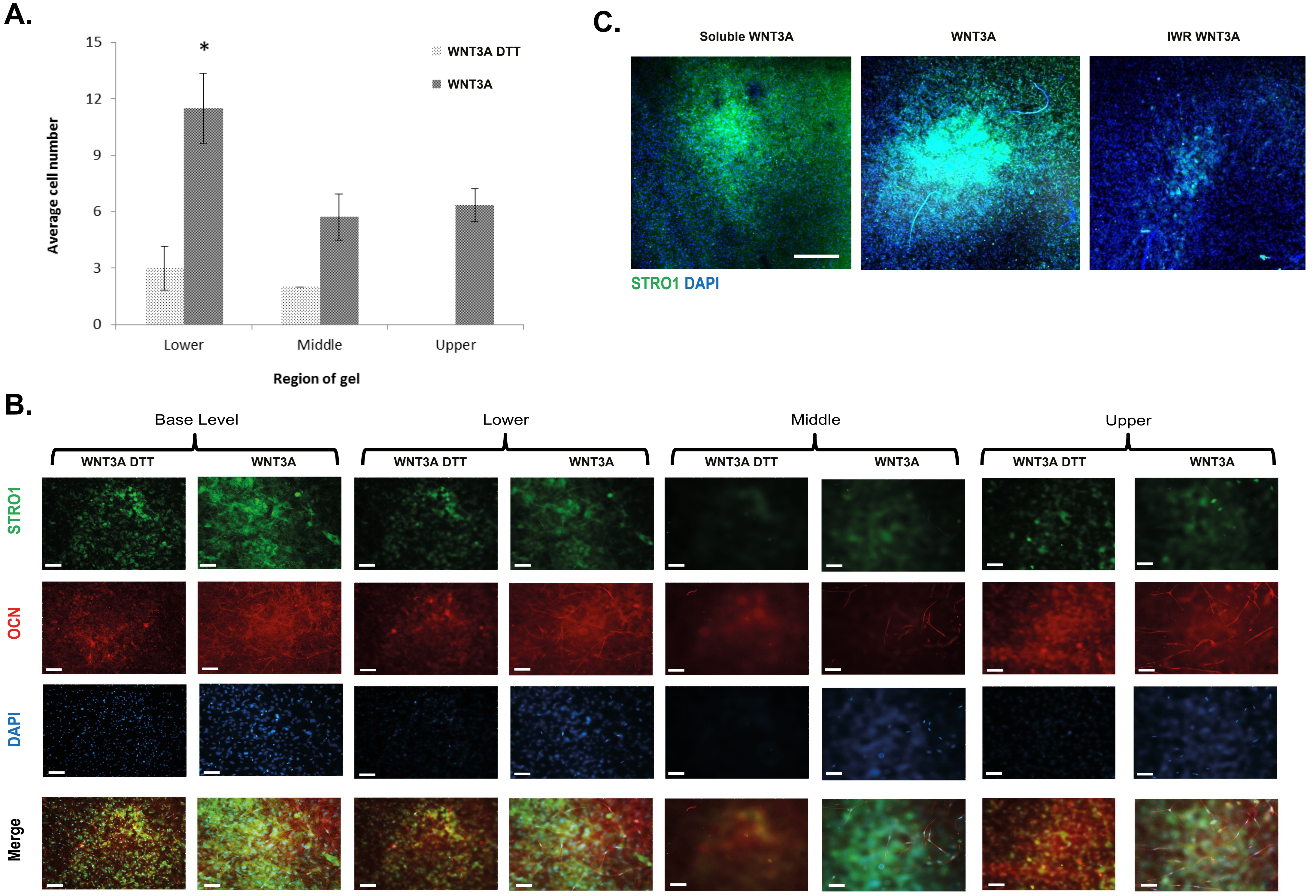

Lowndes et. al. Supplemental Figure 4
