# Supplementary material for: Immobilized WNT Proteins Act as a Stem Cell Niche for Tissue Engineering
Source: Stem Cell Reports. 2016 Jul 12;7(1):126–37. doi: 10.1016/j.stemcr.2016.06.004 (PMC4944585; doi:10.1016/j.stemcr.2016.06.004)
Supplement: Document S2. Article plus Supplemental Information [file mmc2.pdf]

Stem Cell Reports
Resource
OPEN ACCESS

Immobilized WNT Proteins Act as a Stem Cell Niche for Tissue Engineering

Molly Lowndes,[1] Michael Rotherham,[2] Joshua C. Price,[1,2] Alicia J. El Haj,[2] and Shukry J. Habib[1,*]
[1]Centre for Stem Cells and Regenerative Medicine, King's College London, London SE1 9RT, UK
[2]Institute for Science and Technology in Medicine, Guy Hilton Research Centre, Keele University, Stoke-on-Trent ST4 7QB, UK
*Correspondence: shukry.habib@kcl.ac.uk
http://dx.doi.org/10.1016/j.stemcr.2016.06.004

SUMMARY

The timing, location, and level of WNT signaling are highly regulated during embryonic development and for the maintenance of adult tissues. Consequently the ability to provide a defined and directed source of WNT proteins is crucial to fully understand its role in tissue development and to mimic its activity in vitro. Here we describe a one-step immobilization technique to covalently bind WNT3A proteins as a basal surface with easy storage and long-lasting activity. We show that this platform is able to maintain adult and embryonic stem cells while also being adaptable for 3D systems. Therefore, this platform could be used for recapitulating specific stem cell niches with the goal of improving tissue engineering.

INTRODUCTION

The WNT signaling pathway has been implicated in cell proliferation, differentiation, migration, morphological changes, and apoptosis. The canonical pathway initiates a signaling cascade through the Frizzled (Fz) transmembrane receptor and cytoplasmic Disheveled (Dsh) protein, which results in the stabilization and subsequent translocation of β-catenin to the nucleus where it associates with the DNA binding protein TCF to regulate transcription of target genes (Stamos and Weis, 2013). Notably, some of the WNT proteins have also been shown to be involved in β-catenin-independent responses (van Amerongen, 2012). Aberrant activation of the WNT pathway is one of the most frequent signaling abnormalities known in human cancers and is therefore an area of intense research (Clevers and Nusse, 2012).

WNT signaling acts in the stem cell niche by maintaining self-renewal ability; however, in specific cell types, it is also involved in lineage commitment. Therefore, these signals have profound use in regenerative medicine and regulating stem cell fate in vitro. WNT molecules are lipid modified (Takada et al., 2006; Willert et al., 2003), making them highly insoluble, and in vivo they likely signal to target cells in a localized manner (Alexandre et al., 2014; Clevers et al., 2014; Farin et al., 2016; Goldstein et al., 2006; van den Heuvel et al., 1989). Currently, researchers use purified soluble WNTs, which are stored in the presence of detergents to maintain activity (Willert et al., 2003). Soluble WNT proteins are added globally to cells, and at high concentrations the detergent becomes cytotoxic. In addition, in serum-free media, the protein shows compromised stability and activity (Fuerer et al., 2010). Therefore, using a soluble source does not allow control over the spatial presentation of the protein for tissue engineering. Unlike soluble WNT, immobilization of the protein onto beads has recently been shown to promote asymmetric division of embryonic stem cells, suggesting a localized source provides a distinct signal to target cells (Habib et al., 2013). This may be a critical difference for short-range signaling activity and cell polarization within a niche. Here we report on a platform that provides a highly stable source of detergent-free active WNT molecules that can act as basal niche cues for adult and embryonic stem cells in monolayer and can contribute to the directional cues for engineering 3D tissues.

RESULTS

Active WNT3A Molecules Can Be Stably Immobilized onto Aldehyde-Coated Surfaces and Induce WNT/β-Catenin Signaling

In a one-step reaction, we covalently immobilized WNT3A molecules onto commercially available aldehyde-functionalized surfaces (Figure 1A). Recombinant WNT3A protein bound effectively to the aldehyde surface with on average 76% of the protein remaining on the surface, compared with only 33% of its carrier protein BSA (Figure 1B). From this we calculated the average number of molecules per square millimeter immobilized onto the surface. We estimate that with our method of adding 20 ng of WNT3A protein onto a circle with a diameter of 9 mm, 4×10^9 WNT3A molecules/mm^2 are immobilized onto the aldehyde surface. In all experiments, the amount of WNT3A is reported as the input amount. Incubation of WNT surfaces under cell-culture conditions showed negligible amounts of protein release as shown by immunoblot (Figure 1C). To determine if the immobilized WNT3A remained biologically active on the surface, we seeded a TCF-luciferase reporter cell line (LS/L) onto the surfaces (Habib et al., 2013). LS/L cells showed a dose-dependent response to increasing

This is an open access article under the CC BY license (http://creativecommons.org/licenses/by/4.0/).

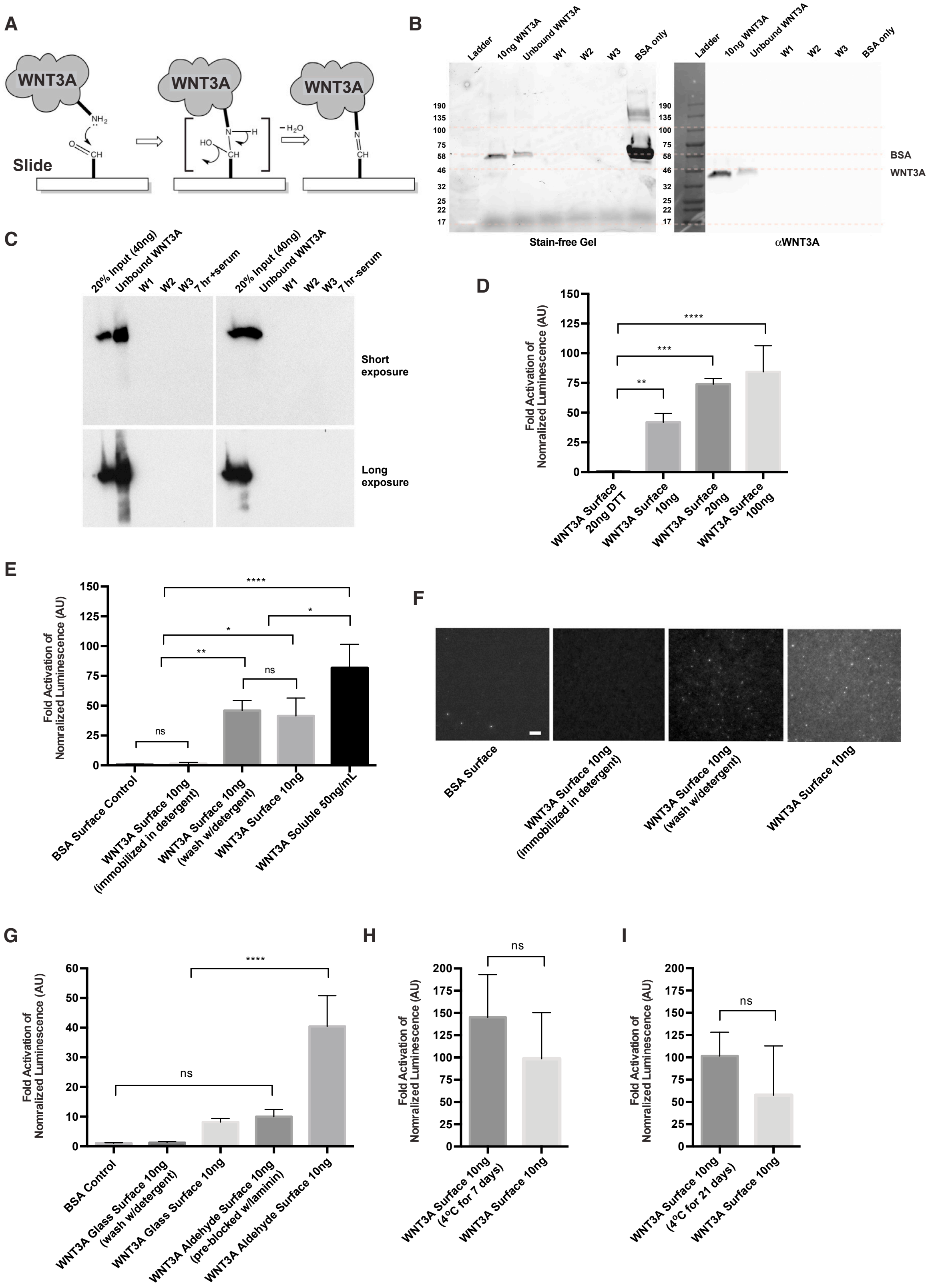

(legend continued on next page)

amounts of WNT3A on the surface, which were all significantly higher than a surface inactivated by treatment with DTT (breaks the crucial disulfide bonds in WNT3A; Habib et al., 2013) (Figure 1D). This method of immobilization in low levels of detergent (0.006%) resulted in better WNT3A activity when compared with a WNT3A immobilized in a high-detergent concentration (1%) and was similar to surfaces incubated with a 1% detergent solution to wash aggregate proteins (Figure 1E). We also visualized the protein using immunofluorescence staining of the surface, comparing the different immobilization techniques (Figure 1F). The covalent immobilization appears to be specific to the aldehyde surface because in non-covalent binding controls, such as pre-blocking the aldehyde groups with laminin protein or incubation on a glass surface, the activity level was not significantly different from control BSA surfaces (Figure 1G). In addition, we found that LS/L cells grown on freshly immobilized WNT3A slides or slides stored at 4°C had a similar activity compared with control surfaces (Figures 1H and 1I). Overall, we suggest that this WNT platform improves the long-term sustainability of the WNT signaling potential while providing a localized basal source to cells.

Immobilized WNT3A Surfaces Can Be Used to Induce WNT Signaling in a Variety of Stem Cell Cultures

To determine if stem cell cultures respond to the immobilized WNT3A surface, we seeded adult mammary gland progenitor cells (Comma Dβ-Geo) (Deugnier et al., 2006) or embryonic stem cells (ESCs) and assayed the WNT/β-catenin response. Using a stable 7xTCF-GFP/SV40-mCherry reporter (Fuerer and Nusse, 2010), we show that Comma Dβ-Geo cells are WNT3A responsive with a dose-dependent increase in GFP expression (Figures 2A and 2B). The number of GFP^+ cells was significantly reduced with an additional DTT treatment (Figures 2A and 2B). In addition, Comma Dβ-Geo cells maintained their responsiveness when seeded onto immobilized WNT3A surfaces stored at 4°C for 2 weeks (Figure S1E) as well as 4 months (Figure S1A). The number of WNT-responsive cells was also confirmed using flow-automated cell sorting analysis (Figure S2). Furthermore, we demonstrate that GFP expression was not due to release of proteins into the medium (Figures 1C and S1B) and expression was maintained for 144 hr when kept on the WNT3A surface in culture (Figures S1C and S1Dii). This is in contrast to the addition of soluble WNT3A to culture medium, which dramatically decreased its effect after 48 hr (Figure S1Div). Importantly, without active WNT3A (either on the surface or supplemented daily), the GFP signal was reduced after 24 hr (Figures S1Diii and S1Dv).

Similar to Comma Dβ-Geo, colony analysis of 7xTCF-GFP expressing ESCs (ten Berge et al., 2011) (Figure 2C) showed a significant increase in the number of GFP^+ colonies (Figure 2D). GFP expression was maintained after 48 hr and upon passaging onto freshly printed WNT3A surfaces (Figure S1F).

This induction of GFP expression was also seen with primary human bone-marrow-derived mesenchymal stem cells (hMSCs) harboring the 7TCF-GFP/SV40-mCherry reporter (Figure 2E). The number of GFP^+ cells was significantly higher when seeded onto a WNT3A surface compared with a surface coated with BSA or WNT3A DTT-treated surfaces (Figure 2F). The number of GFP^+ cells was similar to the level of induction when soluble WNT3A was supplemented into the medium (50 ng/ml). Therefore, this

Figure 1. Active WNT3A Molecules Can Be Stably Immobilized onto Aldehyde-Coated Surfaces and Induce WNT/β-Catenin Signaling

(A) Diagram of the one-step chemical reaction to immobilize WNT3A onto an aldehyde-functionalized surface.

(B) Representative stain-free gel (left) and αWNT3A immunoblot (right) of immobilization process showing WNT3A input (10 ng), unbound WNT3A collected after the incubation and three PBS washings (W1, W2, W3) of the surface. 0.1% BSA alone was also loaded on the gel as a control.

(C) Immunoblot of αWNT3A including wash steps after immobilization of WNT3A along with media supernatant after surfaces incubated at 37°C for 7 hr with (left) and without (right) serum. Proteins were precipitated with trichloroacetic acid before loading onto electrophoresis gels. Long exposure (below) included for visualizing low protein levels.

(D) Normalized luciferase activity assay (reported as fold activation) using LS/L cells harboring a 7xTCF-luciferase reporter. Cells seeded onto immobilized WNT3A surfaces at various concentrations with or without inactivation of WNT3A (DTT treatment).

(E) Luciferase activity assay of WNT3A immobilization conditions: BSA (washed with PBS post-immobilization), WNT3A (immobilized in 1% Chaps solution), WNT3A (washed with 1% Chaps solution post-immobilization), WNT3A (washed with PBS post-immobilization) and WNT3A in solution (50 ng/ml).

(F) Surfaces immunostained for αWNT3A. The scale bar represents 10 μm.

(G) Luciferase activity assay comparing WNT3A immobilization onto different surfaces: glass (washed with 1% Chaps solution post-immobilization procedure), glass (washed with PBS), aldehyde (pre-blocked with laminin protein), and aldehyde (washed with PBS).

(H and I) Luciferase activity assay comparing freshly immobilized surfaces to surfaces stored at 4°C for 1 week and 21 days.

n = 3 independent experiments, mean ± SD; statistical significance using a one-way ANOVA; *p < 0.05, **p < 0.01, ***p < 0.001, ****p < 0.0001; ns, not significant.

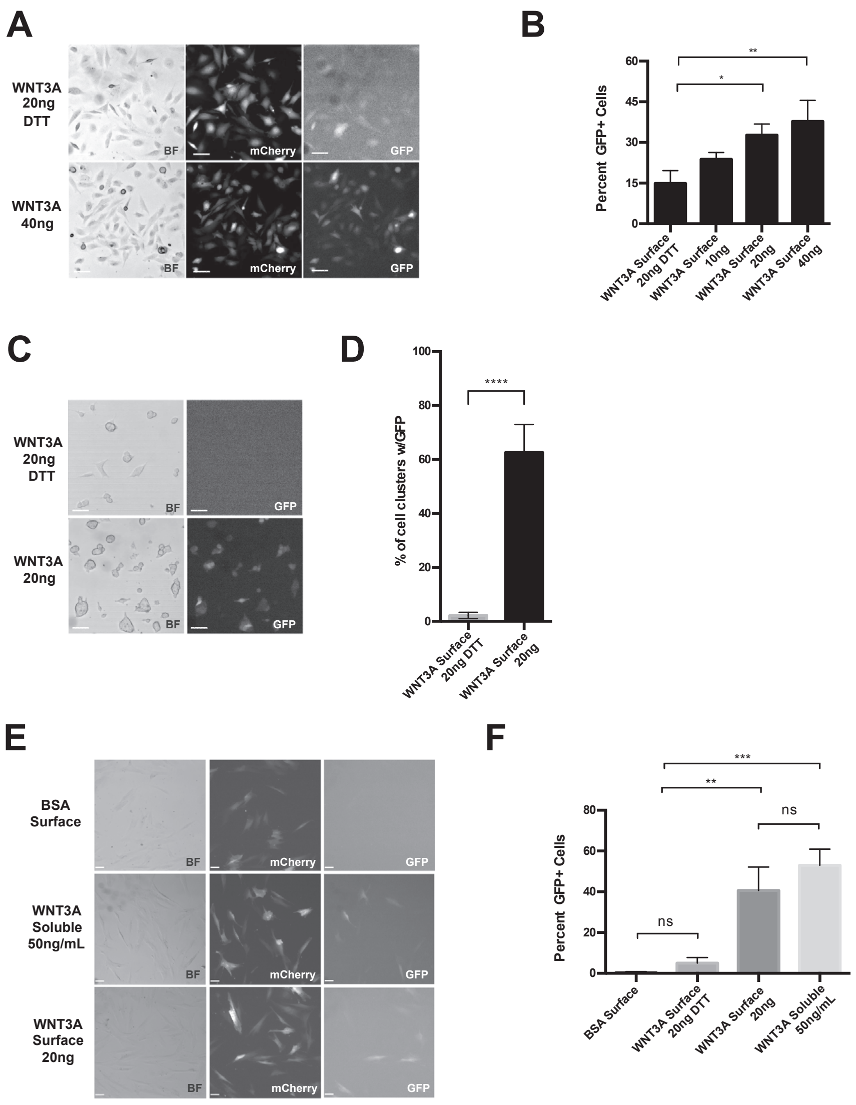

Figure 2. Immobilized WNT3A Surfaces Can Be Used to Induce WNT Signaling in a Variety of Stem Cell Cultures

(A) Brightfield (BF) images of Comma Dβ-Geo cells on WNT3A immobilized surfaces with and without DTT treatment, along with corresponding mCherry (SV-40) and GFP (7xTCF) expression.

(B) Percent GFP^+ cells determined using automated protocol generated in Volocity software; based on finding overlap of mCherry and GFP objects. n = 4 independent experiments, mean ± SD; statistical significance determined with one-way ANOVA, $*p < 0.05$, $**p < 0.01$.

(C) Brightfield images of 7TCF-eGFP mESC colonies and corresponding GFP expression.

(D) Percent of GFP expressing cell clusters on WNT3A or DTT-treated surfaces. Number of colonies determined manually while GFP^+ objects found using automated protocol generated in Volocity software. n = 4 independent experiments, mean ± SD; statistical significance determined by unpaired t test assuming equal SD; $****p < 0.0001$.

(E) Brightfield images of hMSCs expressing 7xTCF-GFP/SV40-mCherry and the corresponding mCherry and GFP expression. Representative images from cells seeded on BSA control, BSA (supplemented with soluble WNT3A), or immobilized WNT3A.

(F) The percent of GFP^+ cells for each condition; determined using automated protocol generated in Volocity software; based on finding overlap of mCherry and GFP objects. n = 3 independent experiments, mean ± SD; statistical significance determined by two-way ANOVA; $**p < 0.01$, $***p < 0.001$; ns, not significant.

The scale bar represents 50 μm.

platform can be used to induce WNT/β-catenin signaling for both embryonic and adult stem cells.

Immobilized WNT3A Surfaces Enrich/Maintain Stem Cell Cultures

Next, as a proof of concept we tested if the WNT3A surface can enrich, without any genetic manipulations, for a population of Comma Dβ-Geo cells expressing progenitor cell markers (SCA1 and purified anti-human/mouse CD49f antibody [integrin α_6]). These progenitors are typically 13%–20% of the total population and can promote outgrowth in cleared fat pad (Chen et al., 2007; Deugnier et al., 2006). Comma Dβ-Geo cells expressing 7TCF-eGFP/mCherry were seeded selectively onto WNT3A surfaces (allowed to adhere for 30 min). To maintain the contact with the surface, 18 hr after seeding cells were stained live on the surfaces for progenitor markers. A representative population of the single-cell maximum expression levels of SCA1 expression shows an increase in population mean compared with DTT-treated surfaces (Figure 3A). When the population was further separated into GFP negative (–), low, and high expression, the mean of the SCA1 population was highest for GFP^{high}-expressing cells (Figures 3B and 3C). Finally, when the cell populations were split into $SCA1^{negative}$ or $SCA1^{positive}$ (based on negative staining controls), the total number of $SCA1^+$ cells on WNT3A surfaces compared with WNT3A DTT surfaces was 69% versus 32%, respectively (Figure 3C). This trend in increased maximum intensity was also seen for integrin α_6 staining (Figures 3D–3F).

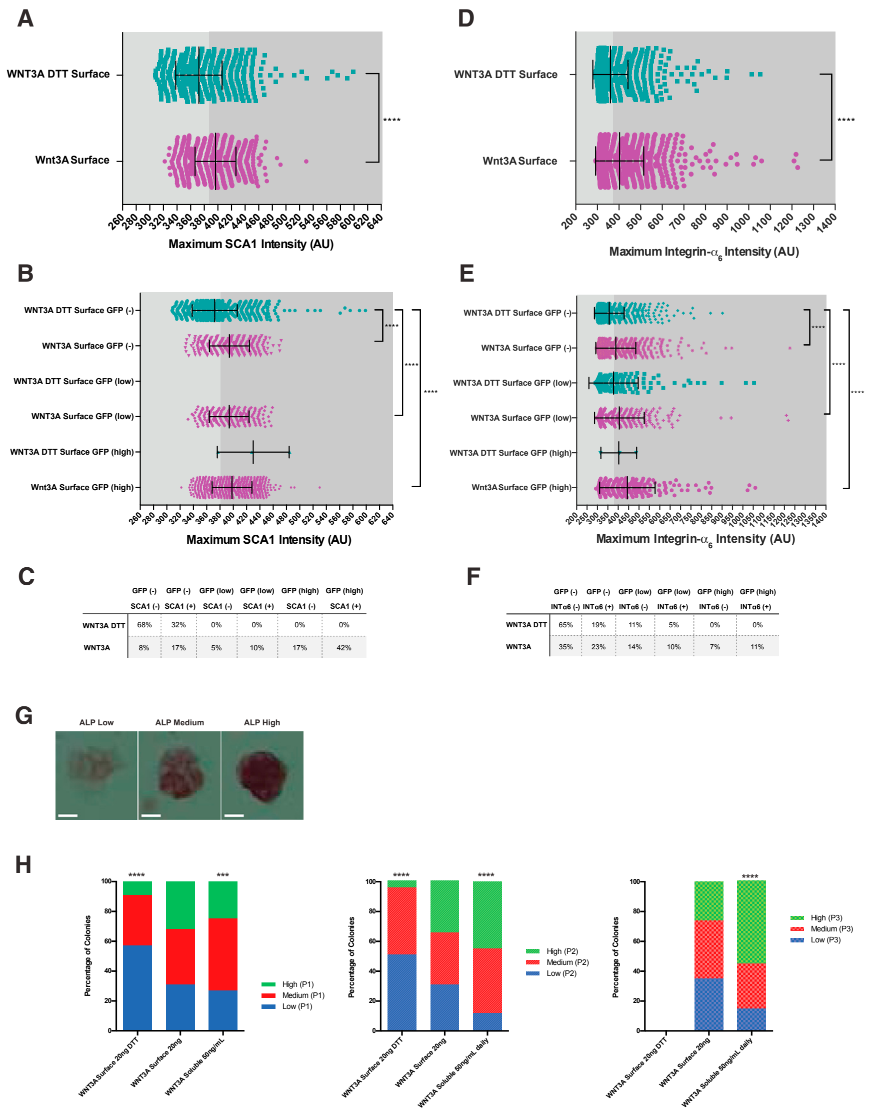

	GFP (-) SCA1 (-)	GFP (-) SCA1 (+)	GFP (low) SCA1 (-)	GFP (low) SCA1 (+)	GFP (high) SCA1 (-)	GFP (high) SCA1 (+)
WNT3A DTT	68%	32%	0%	0%	0%	0%
WNT3A	8%	17%	5%	10%	17%	42%

	GFP (-) INTα6 (-)	GFP (-) INTα6 (+)	GFP (low) INTα6 (-)	GFP (low) INTα6 (+)	GFP (high) INTα6 (-)	GFP (high) INTα6 (+)
WNT3A DTT	65%	19%	11%	5%	0%	0%
WNT3A	35%	23%	14%	10%	7%	11%

Figure 3. Immobilized WNT3A Surfaces Enrich/Maintain Stem Cell Cultures

(A–F) Comma Dβ-Geo cells (7xTCF-GFP/SV40-mCherry) were seeded selectively onto WNT3A DTT or WNT3A surfaces for 30 min. The adherent cells were cultured for 18 hr and then stained live for SCA1 (A–C) or integrin α_6 (D–F). The single-cell maximum expression levels (representative of three independent experiments showing same trend) of SCA1 (A–C) or integrin α_6 (D–F) on immobilized WNT3A or DTT-treated surfaces (A and D). n > 1,000 for each condition; statistical significance determined using Mann-Whitney test (nonparametric); ****$p < 0.0001$. The single-cell spread of the same population when the cells have been separated into (−), (low) or (high) GFP expression (B and E). Statistical significance determined using the Kruskal-Wallis test (nonparametric) comparing distributions to the WNT3A DTT GFP (−) control; ****$p < 0.0001$. Tables summarizing the percentage of cells in each category (C and F).

(G and H) Mouse ESC colonies grown and passaged onto freshly immobilized surfaces (BSA ± soluble WNT3A or WNT3A ± DTT) for up to three passages under serum-free conditions. Between each passage, duplicate wells were stained for alkaline phosphatase (ALP), colonies were counted and split into three categories depending on stain intensity (determined by eye by two researchers); representative color images of three defined levels (scale bar represents 10 μm) (G). Three graphs summarizing the percentages of cells in each category for each passage (H). n = 3 independent experiments; statistical significance determined by comparing the observed population to an expected population equal in each category; ***$p < 0.001$ and ****$p < 0.0001$.

In addition, mean intensity expression levels for SCA1, 7xTCF, and mCherry were visualized with histograms combining single-cell expression from three independent experiments (Figures S3A–S3C). A Tukey boxplot was used to summarize the changes in distribution between the two conditions. The SCA1 mean intensity distribution was significantly different between WNT3A surfaces and DTT-treated surfaces (Kruskal-Wallis test, Kolmogorov-Smirnov D = 0.2007; Figure S3D). The difference in distribution was highly significant for 7xTCF (Kolmogorov-Smirnov D = 0.9387; Figure S3E), while less significant for mCherry (Kolmogorov-Smirnov D = 0.0832; Figure S3F). The percentage of cells in defined categories and the total number of cells analyzed are summarized in Figure S3G. In summary, WNT3A surfaces enrich for WNT-responsive cells, which express the progenitor markers SCA1 and integrin α_6.

Soluble WNT3A, when added to the culture medium daily, was shown to be involved in maintaining the pluripotency of mESCs (ten Berge et al., 2011). We assessed if the immobilized WNT3A platform can maintain this effect. After a 2-day incubation of mESCs on the WNT3A surface with serum-free medium, cells were either trypsinized and passaged onto fresh surfaces or stained for the pluripotency marker alkaline phosphatase (ALP). ALP expression in colonies revealed three distinct populations: low, medium, and high

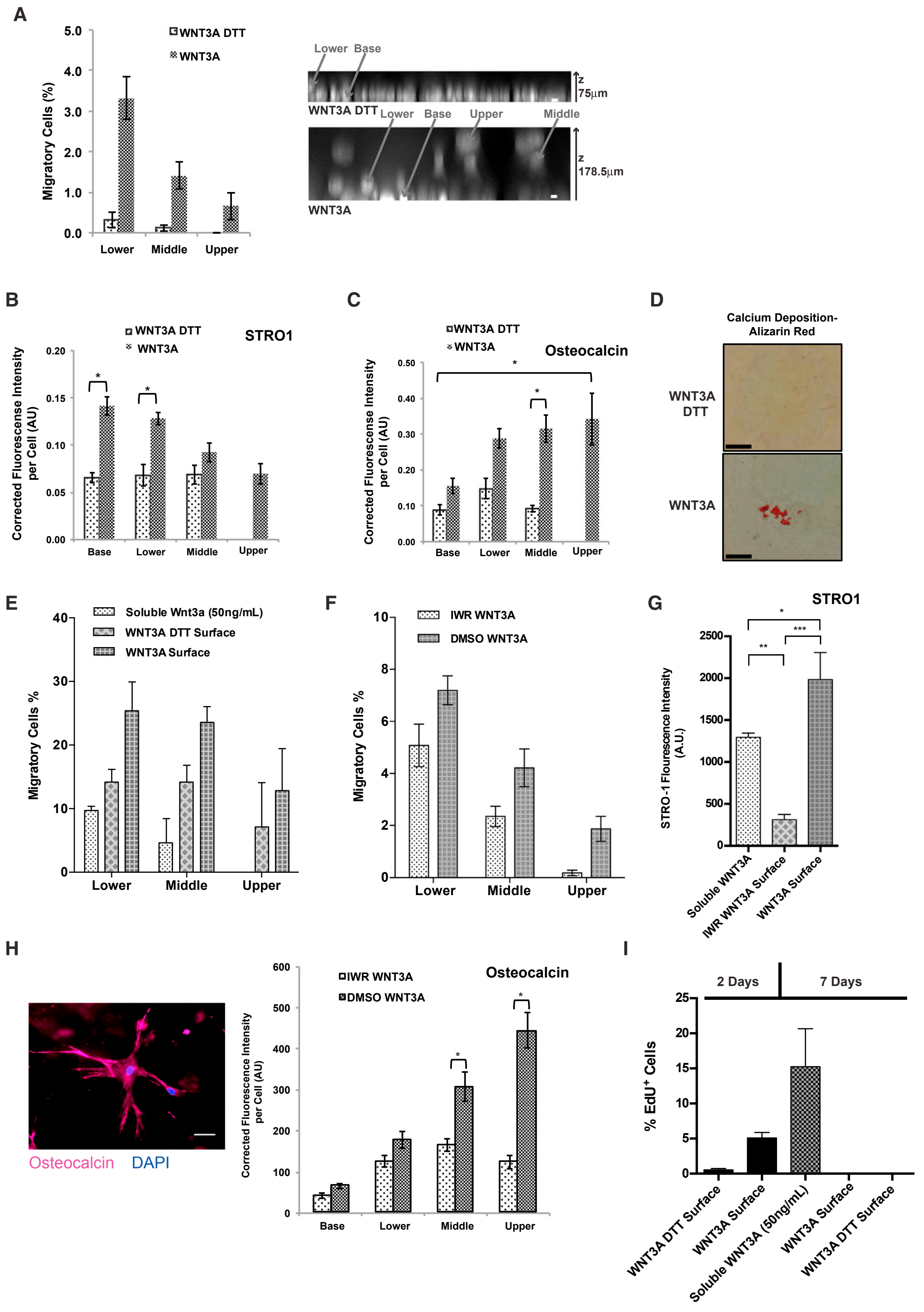

(legend on next page)

expression (Figure 3G). Over three rounds of passaging mESCs showed a consistent distribution of cells in each category, not significantly different when compared with an expected population that is evenly split between the three categories (Figure 3H). This was in contrast to cells grown on DTT-treated surfaces, which mainly had low-expressing ALP colonies and did not survive past passage three. While immobilized WNT3A maintained a heterogeneous population, addition of soluble WNT3A daily resulted in an increase in the proportion of ALP high-expressing colonies over passaging. Similar to the serum-free medium when the cells were grown in the presence of fetal bovine serum (FBS) and passaged, ESCs showed an increase in the proportion of ALP high-expressing colonies compared with DTT-treated surfaces (Figure S3H). WNT3A surfaces also had a greater proportion of colonies expressing high levels of Nanog (28%) compared with DTT-treated surfaces (5%) (Figure S3I). These results suggest that an immobilized WNT3A platform can act as a stable self-renewal cue, potentially improving long-term culturing of WNT-responsive stem cells.

Immobilized WNT3A Surfaces Can Be Adapted to 3D Culture to Direct Human Mesenchymal Stem Cell Differentiation

To test if our methods are applicable for human 3D cell culture, we used primary adult bone-marrow-derived human mesenchymal stem cells (hMSCs), since they are widely thought to represent a clinically relevant source for cell therapies (Wei et al., 2013). It has been previously shown that WNT proteins or active WNT/β-catenin signaling is required for hMSC maintenance in vitro (Boland et al., 2004; de Boer et al., 2004; Ling et al., 2009; Narcisi et al., 2015) and bone formation (Kalajzic et al., 2013; Kramer et al., 2010). As shown in Figure 2F, hMSCs are responsive to a basal WNT signal in 2D culture. To extend our assay to 3D culture, we seeded hMSCs onto immobilized WNT3A surfaces and added an additional spatial dimension by overlaying the cell monolayer with a collagen type I gel. This allowed us to test if basal WNT3A signals could maintain a stem cell population while also assaying for differentiation potential. After only 7 days in culture, we observed an increase in both the number of migratory cells and the distance over which cells migrated into the gel compared with control surfaces (Figures 4A and S4A). Furthermore, we assessed if the 3D model could recapitulate a physiological bone niche by providing oriented WNT signaling to maintain stem cell progeny, while also directing migration, differentiation, and matrix mineralization, analogous to bone turnover by osteoprogenitors in the periosteum (Baron and Kneissel, 2013; Bonewald, 2011; Squier et al., 1990). Therefore, we probed the expression of the osteogenic marker osteocalcin (Chapurlat and Confavreux, 2016) and the mesenchymal stem cell marker STRO1 (Lv et al., 2014; McMurray et al., 2011; Stewart et al.,

Figure 4. Immobilized WNT3A Surfaces Can Be Adapted to 3D-Culture to Control Human Mesenchymal Stem Cell Differentiation

(A) hMSCs were seeded onto immobilized WNT3A ± DTT to form a confluent monolayer before overlaying a collagen gel. After 7 days in culture, cells were fixed and stained with DAPI to mark individual cells. The percentage of cells per layer of the collagen gel (lower level, up to 72 μm from the gel base; middle, 72–132 μm; and upper, 132–179 μm) normalized to the number of cells at the base. A representative bottom-up max projection with DAPI (white) marking each nucleus; example cells in each layer marked with arrows. n = 3 independent experiments, mean ± SEM. The scale bar represents 10 μm.

(B and C) hMSC in collagen gels were fixed after 7 days and immunostained for STRO1 (B) and osteocalcin (C). Expression levels were compared between immobilized WNT3A and DTT-treated. Quantification of normalized (subtracted background) image pixel intensity relative to cell number was plotted. n = 3 independent experiments, mean ± SEM; statistical significance between groups determined by post hoc Mann-Whitney tests; *$p < 0.05$.

(D) Representative histological staining of hMSC gels for calcium deposition (Alizarin red staining) after 7 days of culture. The scale bar represents 100 μm.

(E) hMSCs were grown on immobilized WNT3A ± DTT, BSA ± soluble WNT3A (50 ng/ml). Migrating cells reported as the percentage of total cells in each defined layer of the gel (lower level, up to 80 μm from the gel base; middle, 80–140 μm; and upper, 140–200 μm. n = 3 independent experiments, mean ± SEM.

(F) The percentage of migratory cells in each layer when grown on immobilized WNT3A surfaces ± IWR treatment. n = 3 independent experiments, mean ± SEM.

(G) After 7 days, hMSCs grown on BSA with soluble WNT3A, immobilized WNT3A or immobilized WNT3A with IWR treatment were fixed and immunostained for STRO1. Staining across the middle of the well at the base layer (4× magnification) was quantified. n = 3 independent experiments, mean ± SEM; statistical significance determined by a one-way ANOVA test; *$p < 0.05$, **$p < 0.01$, and ***$p < 0.001$) (for representative confocal images, see Figure S4C).

(H) After 7 days, hMSCs grown on immobilized WNT3A ± IWR treatment were fixed and immunostained for osteocalcin. A representative image of an osteocalcin-expressing cell within the collagen gel (left) and the quantification of osteocalcin in the layers of the collagen gel (right). n = 3 independent experiments, mean ± SEM; statistical significance between groups was determined by post hoc Mann-Whitney tests; *$p < 0.05$. The scale bar represents 50 μM.

(I) Before fixation, 2 days or 7 days with the collagen gel, hMSC cells were stained with EdU and the percentage of EdU^+ cells was quantified. n = 3 independent experiments, mean ± SEM.

1999; Walsh et al., 2001) in our system. We found osteocalcin expression increased as cells migrated upward while STRO1 was highest in the base/lower part of the gel (Figures 4B, 4C, and S4B). To determine if this effect leads to matrix mineralization in the form of calcium deposition, we stained the gels for Alizarin red S and found 2 of 4 WNT3A gels were positive compared with 0 of 4 control gels (Figure 4D). In contrast to immobilized WNT3A, adding soluble WNT3A resulted in reduced migration into the gel while treatment with a WNT/β-catenin signaling inhibitor (IWR) (Chen et al., 2009) reduced but did not block migration (Figures 4E and 4F). At the base layer, STRO1 expression was maintained with the addition of soluble WNT3A, whereas IWR treatment resulted in loss of expression (Figures 4G and S4C). Osteocalcin expression was also lower when treated with IWR (Figure 4H). Finally, to show that the STRO1/osteocalcin gradient was not solely due to cell proliferation, we employed an 5-ethynyl-2′-deoxyuridine (EdU) cell proliferation assay. After 7 days both WNT3A surfaces and DTT-treated surfaces showed no EdU^+ cells, while 15% of the cells grown in the presence of soluble WNT3A (which showed compromised migration) were EdU^+ (Figure 4I). Of note, when the cells were stained after only 2 days with the collagen gel, 5% of the cells on the immobilized WNT3A were EdU^+ (Figure 4I).

Our findings suggest that WNT3A surfaces can act as a stem cell niche, maintaining STRO1 expression at the base and increasing the number of migratory cells that switch expression toward an osteogenic lineage ($osteocalcin^+$). In summary, we propose that our method for immobilizing WNT3A represents a stable platform for more accurately recapitulating a physiological bone niche in vitro.

DISCUSSION

WNT proteins in vivo are often secreted locally and presented to responsive cells in a spatially controlled manner (Alexandre et al., 2014; Clevers et al., 2014; Farin et al., 2016; Goldstein et al., 2006; van den Heuvel et al., 1989). In this study, we present a covalently immobilized WNT platform that can act as a basal signal to support stem cell maintenance and tissue engineering. The aldehyde-amine chemistry does not require the continued presence of detergent and is easier than the multistep immobilization onto the recently published microbeads (Habib et al., 2013). In addition, the dynamics of the culture media makes it challenging to spatially control the WNT microbeads.

The WNT platform can be stored and maintain its signaling activity for a prolonged period. We demonstrate the ability of the basal WNT surfaces to induce WNT/β-catenin signaling as well as enrich and maintain adult stem cells and ESCs in 2D cultures. Unlike soluble WNT3A proteins where ESC passaging increases the proportion of ALP^+ pluripotent colonies, the WNT platform maintains the heterogeneity of ALP colonies in serum-free medium. This system may provide a way to investigate fluctuations between pluripotency/differentiation states of ESCs under defined conditions. We speculate that the non-uniform distribution of immobilized WNT proteins on the surface can be a potential reason for this observation. Alternatively, the division of ESCs on the WNT platform can yield a cell in close proximity to the WNT source, while the daughter cell has less access to the immobilized WNT.

Importantly, the WNT platform can be adapted to 3D tissue culture. To demonstrate this, we used hMSCs, which require WNT signaling for their maintenance and differentiation in 2D culture (Boland et al., 2004; de Boer et al., 2004; Ling et al., 2009; Narcisi et al., 2015). By combining the WNT platform with a 3D culturing system of primary hMSCs, we recapitulated layers of a maturing cell environment of the periosteal bone niche (Bonewald, 2011). Unlike WNT DTT control surfaces, cells in close proximity to the basal WNT platform maintained high expression of the stem cell marker STRO1. In addition, the basal WNT directed migration and differentiation of cells toward an osteogenic phenotype within 7 days of culture. Importantly, adding soluble WNT3A to the 3D system maintained cell proliferation and expression of the stem cell marker STRO1, but migration and differentiation processes were significantly reduced. This implicates the role of spatially confined WNT signals in the maintenance of stem cells and directed cell differentiation. Therefore, spatial presentation of WNT signals to cells in a 3D context can be used for tissue engineering purposes. Our ultimate goal is to mimic basic cellular, signaling, and mechanical (Bonewald and Johnson, 2008; Foster et al., 2015; Robinson et al., 2006; Tu et al., 2012) elements of the bone environment by generating a controlled microsystem of stem cells and a directed differentiation into osteogenic cells in 3D culture.

As many types of stem cells are WNT3A responsive, we anticipate that this platform can be adapted to generate WNT-mediated tissue formation in vitro. Developmental studies show the involvement of other WNTs in tissue patterning (van Amerongen and Nusse, 2009). Fortunately, the predicted protein sequence for the known WNTs shows extensive similarity (Thrasivoulou et al., 2013), suggesting they are likely to be amenable for immobilization using this aldehyde chemistry. With the advances in single-cell analysis and cell population profiling, the proposed WNT platform provides a unique opportunity to further investigate many aspects of localized signaling including the transcriptome and proteome of WNT-responsive cells. Such

analysis in vivo is challenging, as it is difficult to visualize WNT proteins in mammalian systems and correlate the timing of the cellular response. This basic understanding is crucial for studying development and improving cell-based therapy.

The adaptability of the WNT platform allows for the possibility of patterning (Campbell et al., 2005; Ito et al., 2001; Whitesides et al., 2001) the WNT protein and immobilizing it onto a variety of materials that have been functionalized with aldehyde groups. This will allow for potential in vitro analysis of co-cultures when in contact with defined amounts and geometries of WNT signals. In addition, this could improve how the WNTs are presented in 3D culture systems.

Overall, we have characterized a WNT platform that can be used to address fundamental biological questions, facilitate stem cell maintenance, and recapitulate an in vivo stem cell niche in vitro, thus improving the physiological relevance of such systems for developing tissue engineering strategies.

EXPERIMENTAL PROCEDURES

Functionalization of Aldehyde Surfaces

Recombinant mouse WNT3A proteins were produced in *Drosophila* S2 cells grown in suspension culture and purified by Blue Sepharose affinity and gel filtration chromatography as described (Willert et al., 2003). WNT3A activity was determined in a luciferase reporter assay using L cells stably transfected with the SuperTOPFlash reporter as described (Mikels and Nusse, 2006). Alternatively, WNT3A was purchased from R&D systems and reconstituted in 0.1% BSA (Sigma) to a concentration of 40 ng/μl. This was then diluted in PBS for a final volume of 40 μl per drop to cover a working area of ~64 mm^2 on VSS25 Vantage Silylated Slides (Aldehyde) (CEL Associates) with a range of concentrations from 10 ng to 40 ng of WNT3A protein. WNT proteins were incubated on the slide for 1 hr at room temperature. Control (vehicle) surfaces were immobilized with 0.1% BSA in PBS. Control (inactivated WNT3A) surfaces were incubated for an additional 30 min with 20 mM DTT in H_2O at room temperature. The BSA-functionalized surfaces were also used for the soluble WNT3A control where the medium was supplemented with WNT3A (50 ng/ml) daily. Before seeding cells onto the functionalized surfaces, each area was rinsed at least three times with PBS and incubated with the medium used for seeding the cells for 10 min at room temperature. For control experiments, surfaces were coated with laminin protein (50 μg/ml) prior to incubation with WNT3A protein. Immobilization in higher detergent and additional detergent washing were performed with a 1% Chaps solution (PBS).

Cell Culture and Seeding onto Surfaces

L cells stably transfected with the SuperTOPFlash reporter (LS/L) as previously described (Mikels and Nusse, 2006) were grown in DMEM with 10% FBS (Sigma) and 1% penicillin/streptomycin solution (PS) (Sigma). WNT-induced luciferase activity was determined using the Dual-Light System (Applied Biosystems). Comma Dβ-Geo variant cells stably infected with 7xTCF-GFP/SV40-mCherry (Fuerer and Nusse, 2010) were routinely passaged every 2–3 days in DMEM supplemented with 2% FBS, 1% PS, 10 μg/ml insulin (Sigma), and 5 ng/ml EGF (Rouche). Cells were seeded with 5,000–10,000 cells per working area (~64 mm^2).

Routine culture of R1 mESCs (and R1 cells harboring the 7xTCF-GFP reporter) was carried out in Advanced DMEM/F12, 10% ES Cell Qualified FBS (Millipore), 1% PS, 2 mM GlutaMAX (Life Technologies), 50 μM β-mercaptoethanol (Gibco), and 1,000 U/ml leukemia inhibitory factor (LIF; Miltenyi). mESCs were maintained as small clonal colonies by passaging every 2–3 days and changing medium daily. Cells were seeded onto functionalized surfaces at 5,000–10,000 cells per 64 mm^2 and, when necessary, changed to N2B27 medium (serum-free) after cells had adhered (~4 hr). N2B27 medium comprised one volume of DMEM/F12 and one volume of Neurobasal medium supplemented with 0.5% N2 supplement, 1% B27 supplement, 0.033% BSA 7.5% solution, 50 μM β-mercaptoethanol, 2 mM GlutaMAX, 1% PS, and 1,000 U/ml LIF (ten Berge et al., 2011). For ALP staining experiments, mESC medium was also supplemented with 2 μM IWP2 (Miltenyi) to block the secretion of endogenous WNTs (Chen et al., 2009) and changed daily. After 2–3 days on the surfaces, cells were stained using an ALP detection kit (Millipore).

Fresh human bone marrow (Lonza; catalog no. 1M-125) was sourced from healthy volunteers with written informed consent obtained by Lonza. The adherent cell population was selected from fresh bone marrow by culturing aliquots of aspirate for 2 weeks in low glucose DMEM with 5% FBS, 1% L-glutamine, and 1% PS with medium changes performed once per week. MSCs were routinely characterized for expression of surface markers (CD73+, CD90+, CD105+, and CD45−, CD34−, CD14−, CD19−, and HLA-DR−) and histological staining for osteogenic (Alizarin red), chondrogenic (Alcian blue), and adipogenic differentiation (oil red o) (data not shown). For cell expansion, MSCs were cultured in high glucose DMEM supplemented with 10% FBS, 1% L-glutamine, and 1% PS (all reagents from Lonza). Cells were passaged once per week and cells between passages 2 and 5 were used in all experiments. Cells were seeded onto functionalized surfaces at 80,000 cells/cm^2 and cultured for 24 hr after which 100 μl of 1 mg/ml rat tail collagen 1 (BD Biosciences) pre-neutralized with 1 M NaOH (25 μl per ml of gel) and diluted in serum-free media was laid over the cell monolayer. Samples were incubated for 2 hr at 37°C, 5% CO_2 to induce gel crosslinking. Media were then changed to osteogenic media consisting of basal medium with the addition of dexamethasone (0.1 μM), β-glycerophosphate (B-GP) 10 mM, ascorbic acid (50 μM), and non-essential amino acids (NEAA) 1× v/v. Samples were cultured for 2 or 7 days with two medium changes performed. When grown in the presence of IWR (10 μM) or soluble WNT3A (50 ng/ml), medium was changed daily.

Western Blot, Fluorescence-Activated Cell Sorting, and Immunocytochemistry

To determine protein levels in the input and washes during surface functionalization, we used protein electrophoresis, western blotting, and immunofluorescence. Samples were mixed with 4× Laemmli buffer and loaded onto stain-free gels (Bio-Rad) to

determine BSA levels and then immunoblotted for αWNT3A (Millipore; 09-162) overnight at 4°C in 5% milk in TBST (Tris-buffered saline and Tween 20; 1:1,000) after 1 hr blocking in 5% milk TBST. Bands were visualized with rabbit-horseradish peroxidase (1:3,000) (GE Healthcare; NA934) and chemiluminescence on the ChemiDoc (Bio-Rad). Three independent immobilizations onto the surfaces were used to calculate the average amount of protein bound (as reported in the text). To determine the number of molecules bound on the surface, we used the following information. Input of 0.5 μl of 40 ng/μl WNT3A protein, a predicted molecular weight of 37 kDa, and assuming on average 76% of the WNT3A protein binds to the surface, we can calculate the number of molecules per mm^2 (circle d = 9 mm). We estimate 4×10^9 molecules of WNT3A protein are immobilized per mm^2.

$$\mathrm{WNT3A}\,\frac{\mathrm{molecules}}{\mathrm{mm}^2}=\frac{\mathrm{V\ WNT3A}}{\pi\left(\frac{d}{2}\right)^2}\times[\mathrm{WNT3A}]\times(\%\ \mathrm{bound})\times\frac{1}{\mathrm{kDa}}$$

$$\mathrm{WNT3A}\,\frac{\mathrm{molecules}}{\mathrm{mm}^2}=\frac{0.5\ \mu\mathrm{l}\ \ \mathrm{of\ WNT3A}}{\pi(4.5\ \ \mathrm{mm})^2}\times\frac{40\ \mathrm{ng}}{\mu\mathrm{l}}\times(0.76)\times 6.02\times 10^{23}\frac{\mathrm{molecules}}{37\ \ \mathrm{kg}}$$

Immunofluorescence of functionalized surfaces was determined by blocking with 1% BSA in PBS, followed by incubation with αWNT3A (Millipore; 09-162) overnight at 4°C in block solution (1:250), washed three times with PBS and incubated for 1 hr with donkey anti-rabbit IgG secondary antibody, Alexa Fluor 488 (1:1,000) (Life Technologies; A-21206) in block solution. Samples with low protein levels were precipitated from the aqueous solutions by adding trichloroacetic acid to a final concentration of 12% (w/v). The samples were incubated for 10 min on ice or at −20°C, and then centrifuged (36,700 × *g*, 20 min, 2°C). The precipitated proteins were washed with acetone (kept at −20°C), and re-centrifuged (36,700 × *g*, 10 min, 2°C). The protein pellet was dried at room temperature and dissolved in 2× Laemmli buffer.

Fluorescence-activated cell sorting was performed by harvesting cells with 0.25% trypsin-EDTA solution, filtering, and analyzing using the FACSCanto II system (BD Biosciences) (operated by the King's College Biomedical Research Facilities Flow Cytometry Core). Each population of cells was separated into live single cells using the same gates; cells (FSC-AxSSC-A), live (FSC-WxDapi), and single (FSC-WxFSC-A). Compensation controls were determined using Comma Dβ-Geo cells not expressing the virus construct 7xTCF-GFP/SV40-mCherry. Analysis was performed using FlowJo software.

To stain Comma Dβ-Geo cells live for SCA1, cells were first seeded onto the surfaces for 30 min and washed twice with PBS before adding back growth medium supplemented with 2 μM IWP2 and incubated overnight in normal culture conditions (~18 hr). Cells were then washed twice with PBS and incubated with αSCA1-APC (1:50; eBiosciences; 17-5981-81) or integrin α_6 (1:250; BioLegend; 313602) in PBS with 10% FBS for 20 min on ice followed by goat anti-rat IgM secondary antibody, Alexa Fluor 647 (1:250; Life Technologies; A-21248) in PBS with 10% FBS for 20 min on ice. Cells were washed three times with PBS and returned to normal culture medium during imaging.

To determine calcium deposition, hMSC samples were stained with 1% w/v Alizarin red S solution (in D_2O) for 10 min at room temperature. Samples were then washed for 5 × 5 min with D_2O. For immunocytochemistry of hMSCs, cells were washed with PBS then fixed with 4% PFA (Sigma) in PBS for 10 min. Cells were permeabilized with 0.1% Triton X-100 in PBS (Sigma) for 10 min then blocked with 2% BSA (Fisher) in PBS for 2 hr at room temperature. Cells were then incubated with human STRO1 antibody (R&D Systems; MAB1038) diluted 1:50 in 1% BSA in PBS overnight at 4°C. Samples were washed for 3 × 5 min with PBS before incubation with anti-mouse IgG-FITC antibody produced in goat (Sigma; F0257) 1:1,000 in 1% BSA in PBS for 1 hr at room temperature. Samples were washed for 3 × 5 min with PBS. This procedure was repeated for osteocalcin staining where cells were re-blocked with 2% BSA in PBS for 2 hr before incubation with human/rat osteocalcin antibody (R&D Systems; MAB1419) diluted 1:1,000 in 1% BSA in PBS overnight at 4°C. Cells were washed for 3 × 5 min with PBS before incubation with donkey anti-mouse IgG secondary antibody, Alexa Fluor 647 (Life Technologies; A-31571) 1:2,000 in 1% BSA in PBS for 1 hr at room temperature. Samples were washed for 3 × 5 min with PBS and counterstained with DAPI (Sigma) diluted to 1 μg/ml in PBS for 10 min at room temperature. DAPI solution was aspirated and samples were stored in PBS at 4°C before imaging.

Imaging and Analysis

Western blots were analyzed using ImageJ software to determine protein levels. Immunofluorescence of functionalized surfaces was visualized with a Nikon Eclipse TS100 fluorescence scope with a Hamamatsu ORCA-05G camera and NIS-Elements D software. ALP staining was visualized with a Nikon upright Eclipse 80*i* with Digital sight color camera and NIS-Elements software. Expression levels of four independent experiments were determined manually based on darkness and consistency throughout each colony and divided into three categories (low, medium, and high). The % of each category was calculated for each experiment and compared between WNT3A ± DTT and soluble WNT3A. Statistical significance was determined using an expected distribution between the three categories or a one-way ANOVA for comparing percentages of single categories with GraphPad Prism software.

Live-cell analysis of Comma Dβ (7TCF-GFP/SV40-mCherry, SCA1-APC, integrin α_6), mESC (7TCF-GFP), and hMSC (7TCF-GFP/SV40-mCherry) was performed on a Zeiss inverted Axio Imager fluorescence microscope using Zen 2 (Bleu edition) software. Expression levels were determined using Volocity (object finder) or ICY software (spot detector). To determine GFP levels, Comma Dβ-Geo and hMSC cells were first found using mCherry expression and determined to be GFP^+ when above a specific threshold (based on the negative control), while mESCs were found by thresholding GFP levels defined as colonies (clusters of cells) with a mean GFP signal above a particular threshold (based on negative control) determined by automated analysis (ICY object finder) and then counting colonies manually. Significance was determined using a two-way ANOVA test (for multiple comparisons) or an unpaired t test with GraphPad Prism software. For

population analysis of Comma Dβ-Geo cells stained with SCA1 or integrin α_6, images were processed using a protocol designed in ICY software to detect mCherry cells, dilate the region of interest to cover the whole outline of the cell, and measure maximum or mean APC expression. Single cells were also split into three GFP expression categories (based on negative controls). Single-cell expression levels were visualized in GraphPad Prism software and means compared. In addition, the mean expression levels of SCA1 for three independent experiments were visualized with histograms and Tukey boxplots. Statistical analysis by a Kruskal-Wallis test was done after removing outliers (Q = 0.1%) from each individual experiment. The differences in the shape of the two distributions were also analyzed with a Kolmogorov-Smirnov test, with the D number reported for each marker.

Migration in the collagen gel was determined using Z stacks of hMSCs obtained on a Zeiss inverted Axio Imager fluorescence microscope using Zen 2 (Bleu edition) software with a step size of 1.5 μm obtained across a total of 8–12 regions from three gels for each condition. The gel was separated into three layers in the z dimension, a lower layer constituting the lower 46% (up to 72 μm) from the gel base, a middle layer constituting up to 85% (up to 132 μm) from the gel base, and an upper layer constituting up to 100% (up to 179 μm) from the gel base. The total number of cells at the base layer was used to normalize the number of migrating cells for each region and the average percentage/cell number per gel layer was reported with the SE. For immunofluorescence of STRO1 and osteocalcin, the expression levels are reported as background-corrected average staining intensity per cell for the base layer and the three identified migration layers (determined using ImageJ, v1.48s). The cell migration per gel layer was assessed using one-way ANOVA with post hoc Tukey tests with statistical significance at 95% confidence level determined using Mini-tab (v16). Osteocalcin and STRO1 staining intensity were assessed using the Kruskal-Wallis test with statistical significance at the 95% confidence level. Post hoc Mann-Whitney tests were used to determine statistically significant differences between groups. Confocal imaging of STRO1 staining at the base layer was visualized with an Olympus TBI-U90 inverted laser scanning confocal microscope at 4× magnification.

SUPPLEMENTAL INFORMATION

Supplemental Information includes four figures and can be found with this article online at http://dx.doi.org/10.1016/j.stemcr.2016.06.004.

AUTHOR CONTRIBUTIONS

S.J.H. conceived the project. M.L. and S.J.H. organized and wrote the manuscript; M.L., M.R., J.P., and S.J.H. contributed to experiments. All authors contributed to data analysis, discussed the results, and commented on the manuscript.

ACKNOWLEDGMENTS

We thank Dr Roel Nusse for his comments on the experiments and support. We appreciate the assistance of the Biomedical Research Centre Flow Core. This work was supported in part by a Sir Henry Dale Fellowship (S.J.H.) and a grant from UK Regenerative Medicine Platform (S.J.H. and A.J.E.).

Received: December 13, 2015
Revised: June 7, 2016
Accepted: June 8, 2016
Published: July 12, 2016

REFERENCES

Alexandre, C., Baena-Lopez, A., and Vincent, J.-P. (2014). Patterning and growth control by membrane-tethered Wingless. Nature *505*, 180–185.

Baron, R., and Kneissel, M. (2013). WNT signaling in bone homeostasis and disease: from human mutations to treatments. Nat. Med. *19*, 179–192.

Boland, G.M., Perkins, G., Hall, D.J., and Tuan, R.S. (2004). Wnt 3a promotes proliferation and suppresses osteogenic differentiation of adult human mesenchymal stem cells. J. Cell Biochem. *93*, 1210–1230.

Bonewald, L.F. (2011). The amazing osteocyte. J. Bone Miner. Res. *26*, 229–238.

Bonewald, L.F., and Johnson, M.L. (2008). Osteocytes, mechanosensing and Wnt signaling. Bone *42*, 606–615.

Campbell, P.G., Miller, E.D., Fisher, G.W., Walker, L.M., and Weiss, L.E. (2005). Engineered spatial patterns of FGF-2 immobilized on fibrin direct cell organization. Biomaterials *26*, 6762–6770.

Chapurlat, R.D., and Confavreux, C.B. (2016). Novel biological markers of bone: from bone metabolism to bone physiology. Rheumatology (Oxford). http://dx.doi.org/10.1093/rheumatology/kev410.

Chen, M.S., Woodward, W.A., Behbod, F., Peddibhotla, S., Alfaro, M.P., Buchholz, T.A., and Rosen, J.M. (2007). Wnt/beta-catenin mediates radiation resistance of Sca1+ progenitors in an immortalized mammary gland cell line. J. Cell Sci. *120*, 468–477.

Chen, B., Dodge, M.E., Tang, W., Lu, J., Ma, Z., Fan, C.-W., Wei, S., Hao, W., Kilgore, J., Williams, N.S., et al. (2009). Small molecule-mediated disruption of Wnt-dependent signaling in tissue regeneration and cancer. Nat. Chem. Biol. *5*, 100–107.

Clevers, H., and Nusse, R. (2012). Wnt/β-catenin signaling and disease. Cell *149*, 1192–1205.

Clevers, H., Loh, K.M., and Nusse, R. (2014). Stem cell signaling. An integral program for tissue renewal and regeneration: Wnt signaling and stem cell control. Science *346*, 1248012.

de Boer, J., Siddappa, R., Gaspar, C., van Apeldoorn, A., Fodde, R., and van Blitterswijk, C. (2004). Wnt signaling inhibits osteogenic differentiation of human mesenchymal stem cells. Bone *34*, 818–826.

Deugnier, M.-A., Faraldo, M.M., Teulière, J., Thiery, J.P., Medina, D., and Glukhova, M.A. (2006). Isolation of mouse mammary epithelial progenitor cells with basal characteristics from the Comma-Dbeta cell line. Dev. Biol. *293*, 414–425.

Farin, H.F., Jordens, I., Mosa, M.H., Basak, O., Korving, J., Tauriello, D.V.F., de Punder, K., Angers, S., Peters, P.J., Maurice, M.M., and Clevers, H. (2016). Visualization of a short-range Wnt gradient in the intestinal stem-cell niche. Nature *530*, 340–343.

Foster, N.C., Henstock, J.R., Reinwald, Y., and El Haj, A.J. (2015). Dynamic 3D culture: models of chondrogenesis and endochondral ossification. Birth Defects Res. C Embryo Today *105*, 19–33.

Fuerer, C., and Nusse, R. (2010). Lentiviral vectors to probe and manipulate the Wnt signaling pathway. PLoS One *5*, e9370.

Fuerer, C., Habib, S.J., and Nusse, R. (2010). A study on the interactions between heparan sulfate proteoglycans and Wnt proteins. Dev. Dyn. *239*, 184–190.

Goldstein, B., Takeshita, H., Mizumoto, K., and Sawa, H. (2006). Wnt signals can function as positional cues in establishing cell polarity. Dev. Cell *10*, 391–396.

Habib, S.J., Chen, B.-C., Tsai, F.-C., Anastassiadis, K., Meyer, T., Betzig, E., and Nusse, R. (2013). A localized Wnt signal orients asymmetric stem cell division in vitro. Science *339*, 1445–1448.

Ito, Y., Hayashi, M., and Imanishi, Y. (2001). Gradient micropattern immobilization of heparin and its interaction with cells. J. Biomater. Sci. Polym. Ed. *12*, 367–378.

Kalajzic, I., Matthews, B.G., Torreggiani, E., Harris, M.A., Divieti Pajevic, P., and Harris, S.E. (2013). In vitro and in vivo approaches to study osteocyte biology. Bone *54*, 296–306.

Kramer, I., Halleux, C., Keller, H., Pegurri, M., Gooi, J.H., Weber, P.B., Feng, J.Q., Bonewald, L.F., and Kneissel, M. (2010). Osteocyte Wnt/beta-catenin signaling is required for normal bone homeostasis. Mol. Cell Biol. *30*, 3071–3085.

Ling, L., Nurcombe, V., and Cool, S.M. (2009). Wnt signaling controls the fate of mesenchymal stem cells. Gene *433*, 1–7.

Lv, F.-J., Tuan, R.S., Cheung, K.M.C., and Leung, V.Y.L. (2014). Concise review: the surface markers and identity of human mesenchymal stem cells. Stem Cells *32*, 1408–1419.

McMurray, R.J., Gadegaard, N., Tsimbouri, P.M., Burgess, K.V., McNamara, L.E., Tare, R., Murawski, K., Kingham, E., Oreffo, R.O.C., and Dalby, M.J. (2011). Nanoscale surfaces for the long-term maintenance of mesenchymal stem cell phenotype and multipotency. Nat. Mater. *10*, 637–644.

Mikels, A.J., and Nusse, R. (2006). Purified Wnt5a protein activates or inhibits beta-catenin-TCF signaling depending on receptor context. PLoS Biol. *4*, e115.

Narcisi, R., Cleary, M.A., Brama, P.A.J., Hoogduijn, M.J., Tüysüz, N., ten Berge, D., and van Osch, G.J.V.M. (2015). Long-term expansion, enhanced chondrogenic potential, and suppression of endochondral ossification of adult human MSCs via WNT signaling modulation. Stem Cell Rep. *4*, 459–472.

Robinson, J.A., Chatterjee-Kishore, M., Yaworsky, P.J., Cullen, D.M., Zhao, W., Li, C., Kharode, Y., Sauter, L., Babij, P., Brown, E.L., et al. (2006). Wnt/beta-catenin signaling is a normal physiological response to mechanical loading in bone. J. Biol. Chem. *281*, 31720–31728.

Squier, C.A., Ghoneim, S., and Kremenak, C.R. (1990). Ultrastructure of the periosteum from membrane bone. J. Anat. *171*, 233–239.

Stamos, J.L., and Weis, W.I. (2013). The β-catenin destruction complex. Cold Spring Harb. Perspect. Biol. *5*, a007898.

Stewart, K., Walsh, S., Screen, J., Jefferiss, C.M., Chainey, J., Jordan, G.R., and Beresford, J.N. (1999). Further characterization of cells expressing STRO-1 in cultures of adult human bone marrow stromal cells. J. Bone Miner. Res. *14*, 1345–1356.

Takada, R., Satomi, Y., Kurata, T., Ueno, N., Norioka, S., Kondoh, H., Takao, T., and Takada, S. (2006). Monounsaturated fatty acid modification of Wnt protein: its role in Wnt secretion. Dev. Cell *11*, 791–801.

ten Berge, D., Kurek, D., Blauwkamp, T., Koole, W., Maas, A., Eroglu, E., Siu, R.K., and Nusse, R. (2011). Embryonic stem cells require Wnt proteins to prevent differentiation to epiblast stem cells. Nat. Cell Biol. *13*, 1070–1075.

Thrasivoulou, C., Millar, M., and Ahmed, A. (2013). Activation of intracellular calcium by multiple Wnt ligands and translocation of β-catenin into the nucleus: a convergent model of Wnt/Ca2+ and Wnt/β-catenin pathways. J. Biol. Chem. *288*, 35651–35659.

Tu, X., Rhee, Y., Condon, K.W., Bivi, N., Allen, M.R., Dwyer, D., Stolina, M., Turner, C.H., Robling, A.G., Plotkin, L.I., and Bellido, T. (2012). Sost downregulation and local Wnt signaling are required for the osteogenic response to mechanical loading. Bone *50*, 209–217.

van Amerongen, R. (2012). Alternative Wnt pathways and receptors. Cold Spring Harb. Perspect. Biol. *4*, a007914.

van Amerongen, R., and Nusse, R. (2009). Towards an integrated view of Wnt signaling in development. Development *136*, 3205–3214.

van den Heuvel, M., Nusse, R., Johnston, P., and Lawrence, P.A. (1989). Distribution of the wingless gene product in Drosophila embryos: a protein involved in cell-cell communication. Cell *59*, 739–749.

Walsh, S., Jordan, G.R., Jefferiss, C., Stewart, K., and Beresford, J.N. (2001). High concentrations of dexamethasone suppress the proliferation but not the differentiation or further maturation of human osteoblast precursors in vitro: relevance to glucocorticoid-induced osteoporosis. Rheumatology (Oxford) *40*, 74–83.

Wei, X., Yang, X., Han, Z.-P., Qu, F.-F., Shao, L., and Shi, Y.-F. (2013). Mesenchymal stem cells: a new trend for cell therapy. Acta Pharmacol. Sin. *34*, 747–754.

Whitesides, G.M., Ostuni, E., Takayama, S., Jiang, X., and Ingber, D.E. (2001). Soft lithography in biology and biochemistry. Annu. Rev. Biomed. Eng. *3*, 335–373.

Willert, K., Brown, J.D., Danenberg, E., Duncan, A.W., Weissman, I.L., Reya, T., Yates, J.R., and Nusse, R. (2003). Wnt proteins are lipid-modified and can act as stem cell growth factors. Nature *423*, 448–452.

Stem Cell Reports, Volume *7*

Supplemental Information

Immobilized WNT Proteins Act as a Stem Cell Niche for Tissue Engineering

Molly Lowndes, Michael Rotherham, Joshua C. Price, Alicia J. El Haj, and Shukry J. Habib

Supplemental Figures

Supplementary Figure 1. Long-term storage controls with Comma-β-Geo cells and mESCs, Related to Figure 2

(A) Comma-Dβ-Geo cells seeded onto surfaces immobilized with WNT3A or vehicle. Surfaces were freshly printed or printed then dehydrated and stored at 4°C for 4 months before cell seeding. After 24hrs the number of GFP+ cells per condition determined, summarized in table (below)

(B) Comma-Dβ-Geo cells seeded onto freshly immobilized WNT3A/vehicle or immobilized WNT3A surfaces incubated at 37°C for 7hrs +/- serum. Table below summarizes number of GFP+ cells per condition. The non-bound WNT fractions (from freshly printed or after incubation at 37°C for 7hrs +/- serum) were added to Comma-Dβ-Geo cells seeded onto vehicle surfaces (right).

(C) Representative images of GFP and mCherry expression of Comma-Dβ-Geo cells seeded onto freshly immobilized WNT3A or vehicle surfaces and visualized after 144hrs.

(D) Representative GFP images of Comma-Dβ-Geo cells seeded onto surfaces immobilized with vehicle or WNT3A. An image taken every 24hrs for up to 168hrs and medium was changed every other day. (i) Cells seeded onto vehicle surface. (ii) Cells seeded onto WNT3A surface. (iii) Cells seeded onto vehicle surface after being grown on WNT3A surface for 24hrs. (iv) Cells seeded onto vehicle surface with soluble WNT3A added the first day to the medium. The original medium was kept and further supplemented with a fresh medium without WNT3A (final volume of 200 μl) to keep samples from drying out (v) Cells seeded onto vehicle surface alone

after being grown for 24hrs on vehicle surface with soluble WNT3A supplemented medium.

(E) Percent GFP$^+$ cells on immobilized surfaces stored at 4°C for 14 days (WNT3A ± DTT) determined using automated protocol generated in Volocity software; based on finding overlap of mCherry and GFP objects. (two independent experiments, mean ± SD; statistical significance determined with one-way ANOVA, p values correspond to *<0.05).

(F) The number of GFP$^+$ objects/ESC cell cluster was monitored every 24hrs and upon passaging onto freshly immobilized surfaces. The number of GFP$^+$ objects per cell cluster was significantly different between the two surfaces, (two independent experiments; statistical significance determined with a two-way ANOVA; p values correspond to **<0.01, ***<0.001).

The scale bar represents 50 μM.

Supplementary Figure 2. FACS analysis of WNT responsiveness, Related to Figure 2

FACS analysis of Comma Db-Geo (7x-GFP/SV-40mCherry) cells seeded onto different surfaces for 24hrs.

(A) All conditions were gated for cells (FSC-AxSSC-A), Live (FSC-WxDapi) and single cells (FSC-WxFSC-A).

(B) Control cells used for compensation controls.

(C) Comma Db-Geo (7x-GFP/SV-40mCherry) alone

(D) Comma Db-Geo (7x-GFP/SV-40mCherry) with soluble WNT3A (50ng/mL)

(E) Comma Db-Geo (7x-GFP/SV-40mCherry) seeded onto a BSA coated surface.

(F) Comma Db-Geo (7x-GFP/SV-40mCherry) seeded onto a DTT treated WNT3A surface.

(G) Comma Db-Geo (7x-GFP/SV-40mCherry) seeded onto a WNT3A surface.

Plots are split into 4 quadrants and a FITC-low population is highlighted based on control groups.

Supplementary Figure 3. Additional analysis of marker expression for Comma Dβ-Geo cells and mESCs, Related to Figure 3

(A-F) Single cell fluorescence intensity measurements of Comma Dβ-Geo cells for each marker [SCA1-APC (A and D), 7TCF-GFP (B and E) and SV40-mCherry (C and F)] were combined (three independent experiments) and plotted as a histogram (A-C) and Tukey box plot (D-F) (>1000 cells per experiment with outliers removed from each individual experiment, Q=0.1%). Statistically significant changes in intensity distributions between the two populations were determined using the Kruskal-Wallis test and the Kolmogorov-Smirnov D value is reported.

(G) The expression levels were split into 2-3 categories; corresponding percentages and number of cells analyzed are summarized in a table.

(H) After being grown for two days in ESC medium containing FBS +LIF mESCs on immobilized WNT3A ± DTT or BSA surfaces (supplemented with 50ng/mL soluble WNT3A) ESCs were fixed and stained for alkaline phosphatase (P1) or trypsinized, collected and re-seeded onto freshly immobilized surfaces. After an additional two days the cells were fixed and stained with alkaline phosphatase and quantified (P2). (n=3 biological replicates; statistical significance determined with one-way ANOVA for just the ALP high percentages between surface treatments; p values correspond to *<0.05, **<0.01, ***<0.001)

(I) Representative brightfield and GFP images of Nanog-Venus mESCs grown on WNT3A surfaces (+/- DTT treatment) for 72 hrs. Fluorescence intensity determined using Volocity software and the number of colonies manually. Colonies not above threshold considered Nanog low while colonies above the threshold were considered Nanog high. Population percentages plotted as part of whole for each condition. Colony analysis showed 5% of the colonies on WNT3A DTT surfaces with high Nanog-Venus expression compared to WNT3A surfaces with 28% of the colonies with high Nanog-Venus expression.

Supplementary Figure 4. Raw numbers and representative images of quantified data for hMSC 3D culture, Related to Figure 4

(A) hMSCs cultured on the active WNT3A surfaces coated with collagen gel were stained for DAPI to determine cell number. Gels were imaged as z-stacks and the number of cells in each layer was counted: lower (up to 72µm / 46% gel), middle (up to 132µm, 85% gel) and upper layers (up to 179µm, 100% gel). Values represent average cell counts, error bars represent SEM, * denotes $p<0.05$.

(B) Representative fluorescent immunostaining images from each defined layer. Gels stained for STRO1 (green), Osteocalcin (red) and DAPI (blue) of hMSC in collagen gels after 7 days in culture on WNT3A ± DTT. Merged images show spatial pattern of staining in relation to cell nuclei. Scale bar represents 100 µm.

(C) Representative confocal images (4x magnification) of STRO1 staining at the base of the collagen gel (7 days in culture) in the middle of the well. The scale bar represents 500 µm.

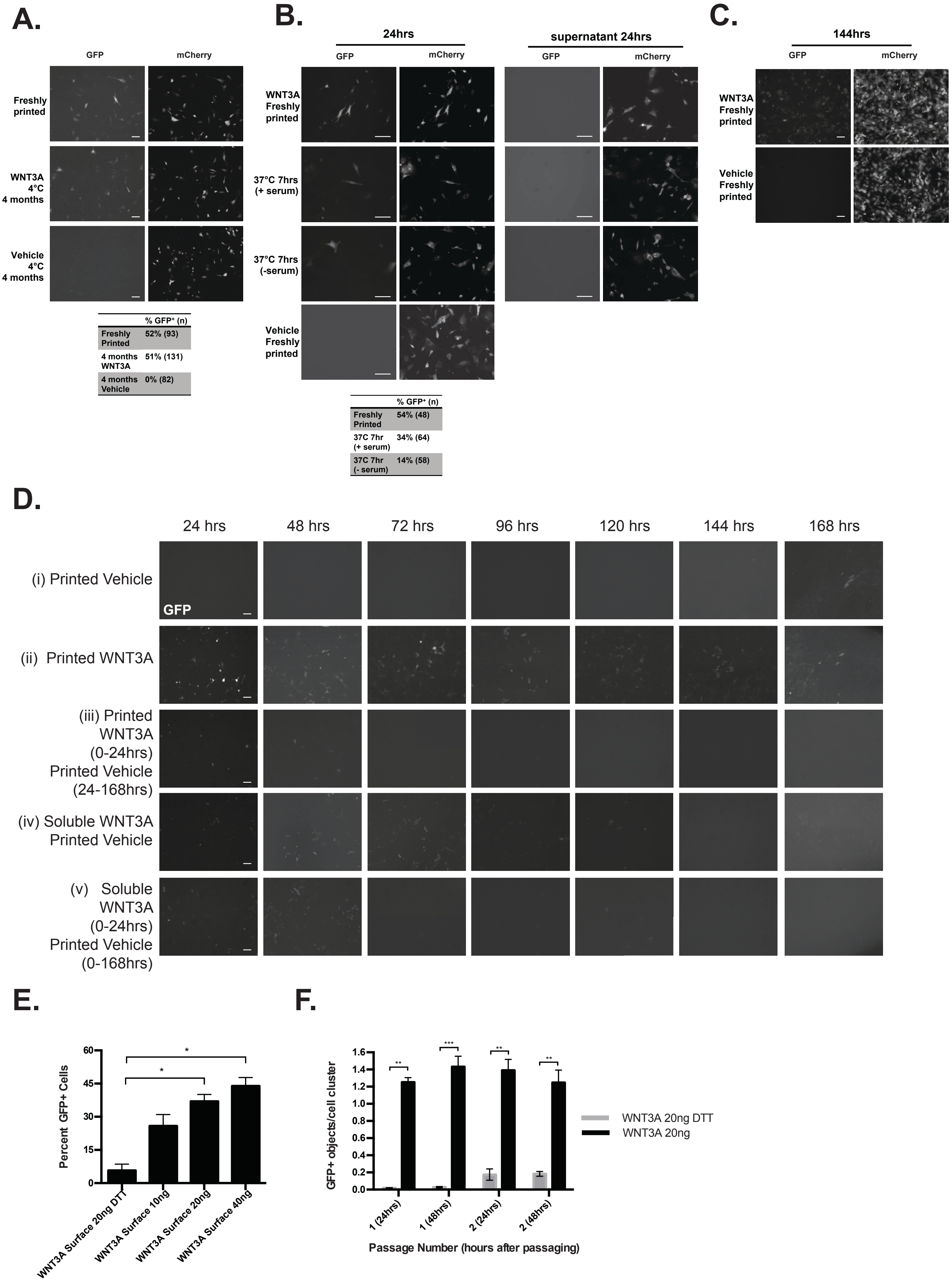

	% GFP+ (n)
Freshly Printed	52% (93)
4 months WNT3A	51% (131)
4 months Vehicle	0% (82)

	% GFP+ (n)
Freshly Printed	54% (48)
37C 7hr (+ serum)	34% (64)
37C 7hr (- serum)	14% (58)

Lowndes et. al. Supplemental Figure 1

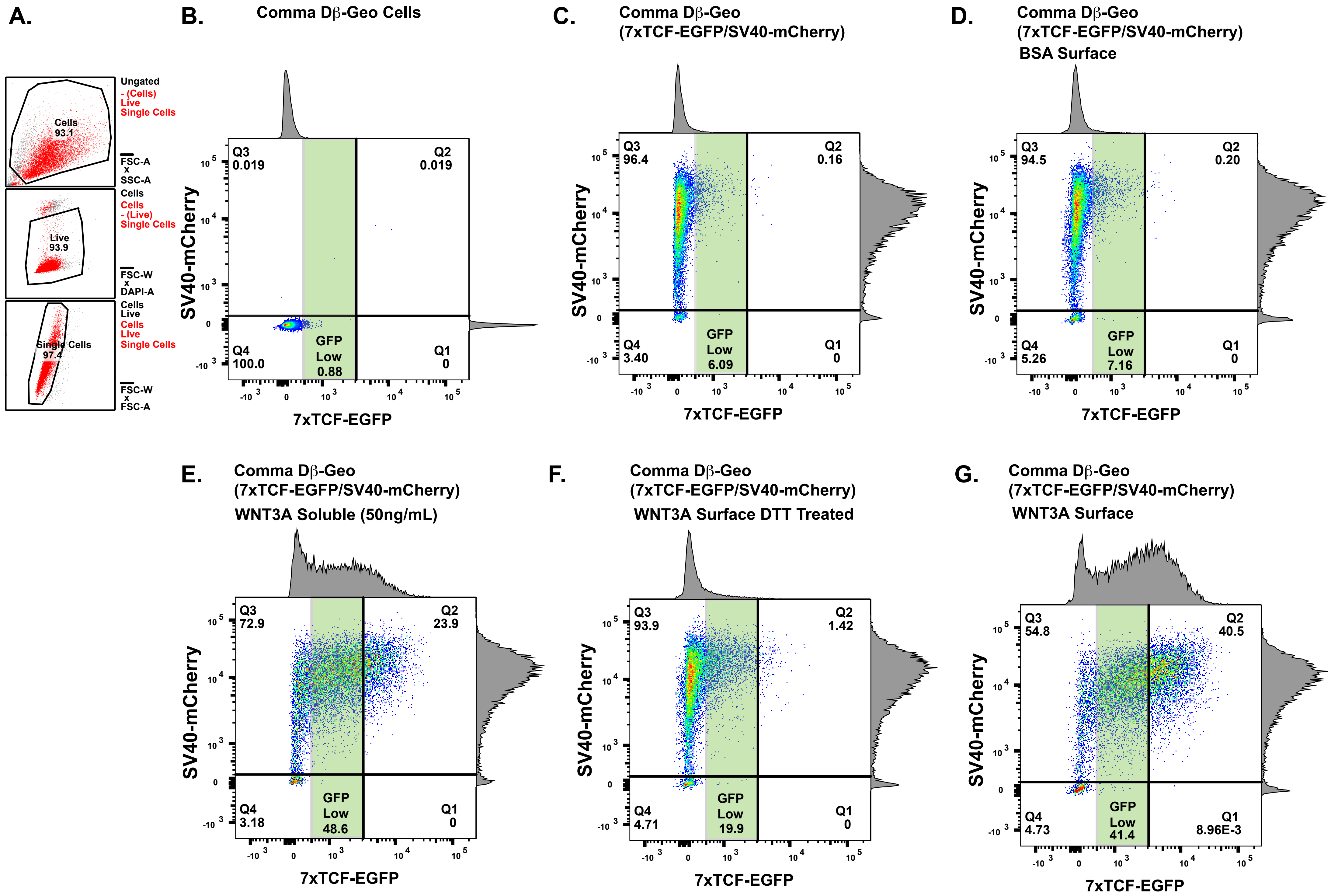

Lowndes et. al. Supplemental Figure 2

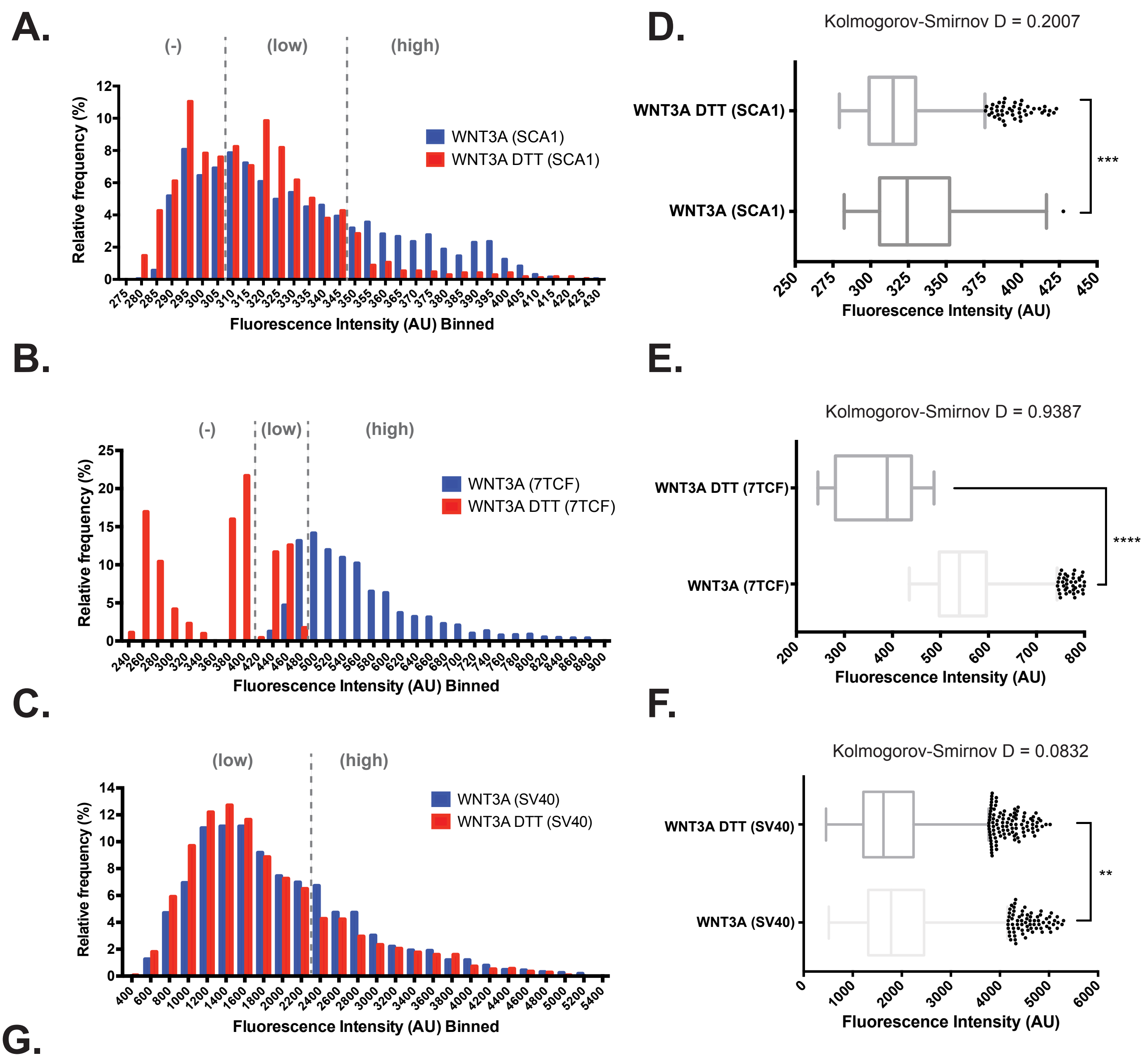

G.

	SCA1 (-)	SCA1 (low)	SCA1 (high)	n
WNT3A DTT	38%	53%	9%	1683
WNT3A	27%	45%	28%	1906

	7TCF (-)	7TCF (low)	7TCF (high)	n
WNT3A DTT	74%	26%	0%	1439
WNT3A	0%	23%	77%	1597

	SV40 (low)	SV40 (high)	n
WNT3A DTT	81%	19%	2877
WNT3A	77%	23%	3166

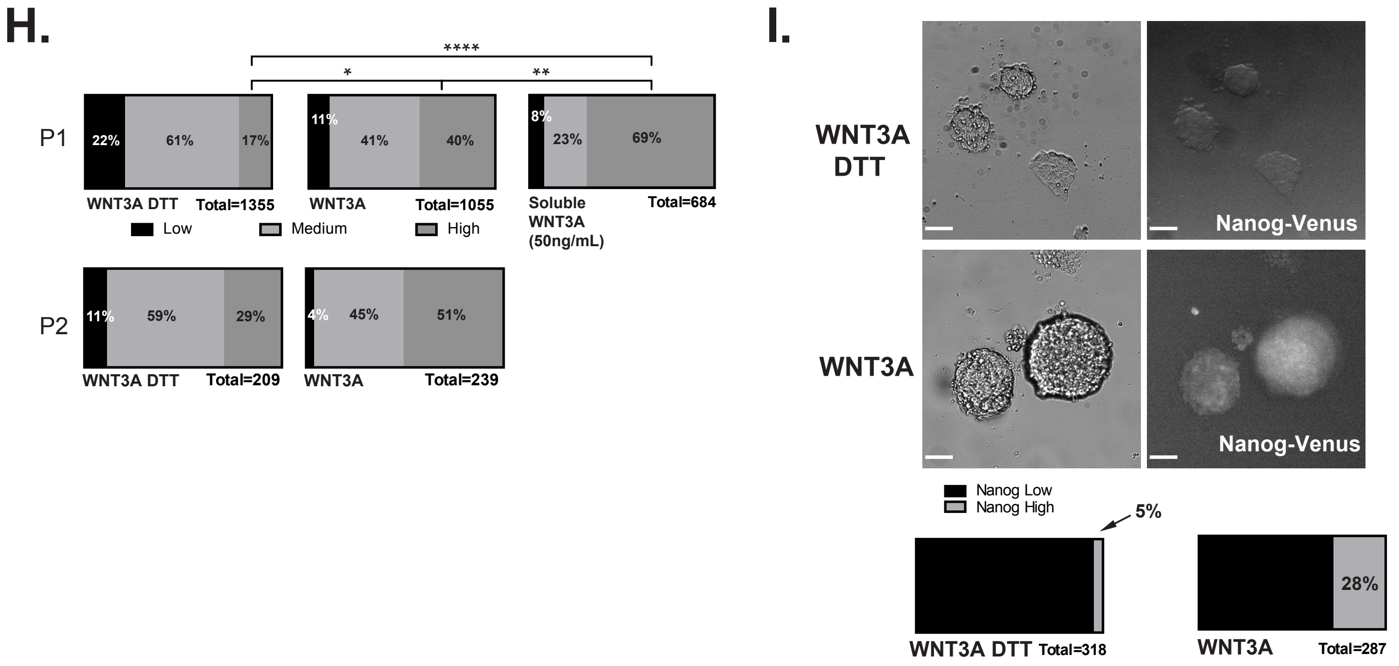

Lowndes et. al. Supplemental Figure 3

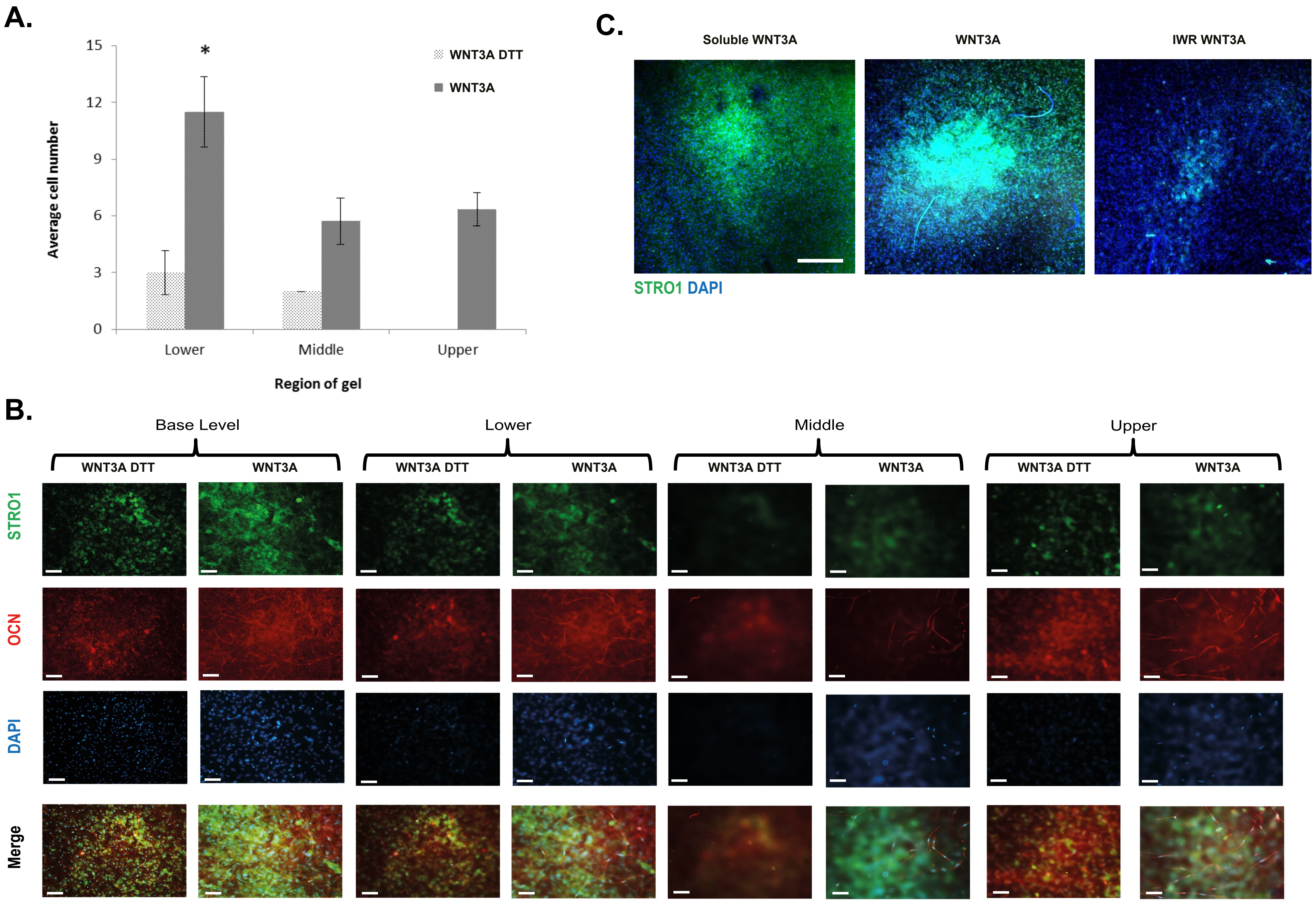

Lowndes et. al. Supplemental Figure 4
